# A Dual Approach with Organoid and CRISPR Screening Reveals ERCC6 as a Determinant of Cisplatin Resistance in Osteosarcoma

**DOI:** 10.1002/advs.202500632

**Published:** 2025-06-06

**Authors:** Ruiling Xu, Sai Zhu, Wenchao Zhang, Haodong Xu, Chao Tu, Honghui Wang, Lu Wang, Na He, Tang Liu, Xiaoning Guo, Xiaolei Ren, Zhihong Li

**Affiliations:** ^1^ Department of Orthopedics The Second Xiangya Hospital Central South University Changsha Hunan 410011 P. R. China; ^2^ Hunan Key Laboratory of Tumor Models and Individualized Medicine The Second Xiangya Hospital Changsha Hunan 410011 P. R. China; ^3^ College of Biology Hunan University Changsha 410082 P. R. China

**Keywords:** alternative splicing, chemoresistance, ERCC6, organoid, osteosarcoma

## Abstract

Osteosarcoma (OS), the most prevalent primary bone malignancy in adolescents, is typically treated with cisplatin‐based chemotherapy. However, the development of cisplatin resistance often leads to relapse or metastasis, significantly impairing therapeutic efficacy. To tackle this issue, patient‐derived osteosarcoma organoids (OSOs) is established that accurately reflect the cellular composition and heterogeneity of the original tumors, as validated by single‐cell RNA sequencing, bulk RNA sequencing, and histopathology analysis. Cisplatin resistance is successfully induced in these OSOs, creating a clinically relevant model for investigating chemoresistance. Utilizing RNA sequencing in cisplatin‐resistance OSOs and CRISPR screening in OS cell line, ERCC6 is identified as a pivotal regulator of cisplatin resistance. Knockdown of ERCC6 markedly enhanced cisplatin sensitivity in vitro and in vivo. Mechanistically, ERCC6 interacts with HNRNPM, influencing the PI3K/AKT signaling pathway and alternative splicing of pre‐mRNA for *BAX*. Notably, the knockdown of ERCC6 and HNRNPM increased expression of full‐length *BAX* and reduced skipping of exon 2, thus promoting apoptosis. This exon skipping in BAX results in a frameshift and introduces a premature stop codon (TGA) within the BH3 domain. These findings underscore the utility of OSOs in elucidating resistance mechanisms and highlight ERCC6 and HNRNPM as potential therapeutic targets.

## Graphical abstract

This study utilizes patient‐derived osteosarcoma organoids and CRISPR screening to identify ERCC6 and HNRNPM as key mediators of cisplatin resistance via the PI3K/AKT pathway and *BAX* alternative splicing. These findings provide novel insights and potential therapeutic targets for overcoming chemoresistance in osteosarcoma. Overall, this work advances personalized oncology.

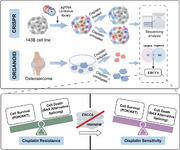

## 1 Introduction

Osteosarcoma (OS) is the most common primary malignant bone tumor in adolescents with a poor prognosis.^[^ ^1^ ^]^ Cisplatin‐based chemotherapy is the major treatment besides surgery.^[^ ^2^, ^3^ ^]^ However, resistance to standard chemotherapy drugs makes chemoresistance a large contributing factor to failed OS treatment,^[^ ^4^ ^]^ resulting in relapse or metastasis and a 5‐year overall survival of less than 30%.^[^ ^5^ ^]^ Despite extensive research on mechanisms of cisplatin resistance in OS, the definitive pathways remain poorly understood. Understanding these mechanisms of drug resistance is essential for creating targeted treatments for patients with resistant OS, leading to improved prognosis.^[^ ^6^, ^7^ ^]^

Historically, models of acquired drug resistance have been constructed by inducing resistance in OS cell lines through in vitro drug exposure, followed by drug screening and target discovery.^[^ ^8^, ^9^ ^]^ However, the limitations of cell line models have resulted in a low clinical translation rate.^[^ ^10^ ^]^ Osteosarcoma organoid (OSO), as a novel in vitro model derived from tumor tissues of OS patients, can be stably cultured and passaged in vitro, effectively mimicking the biological characteristics of OS patient tissues.^[^ ^11^, ^12^ ^]^ The induced drug resistance model based on tumor organoids better simulates the clinical evolution of drug resistance.^[^ ^13^ ^]^ Previously, there has been a lack of studies inducing acquired drug resistance in OSOs in vitro and subsequently exploring the underlying resistance mechanisms and therapeutic targets.^[^ ^14^ ^]^

We developed a method for culturing OSOs and successfully induced cisplatin resistance in these models. Our findings revealed that cisplatin treatment significantly upregulates ERCC6 expression in OSOs, which correlates with the clinicopathological features of osteosarcoma patients. The knockdown of ERCC6 in OS cell lines enhanced their sensitivity to cisplatin and promoted apoptosis. Furthermore, we discovered that ERCC6 interacts with HNRNPM, thereby activating the PI3K/AKT pathway and regulating alternative splicing of BAX pre‐mRNA, which in turn enhances cisplatin resistance in OS cells. These results suggested that ERCC6 and HNRNPM could serve as potential therapeutic targets for overcoming cisplatin resistance in OS.

## 2 Results

### 2.1 Establishment and Characterization of OSOs

**Figure 1:**
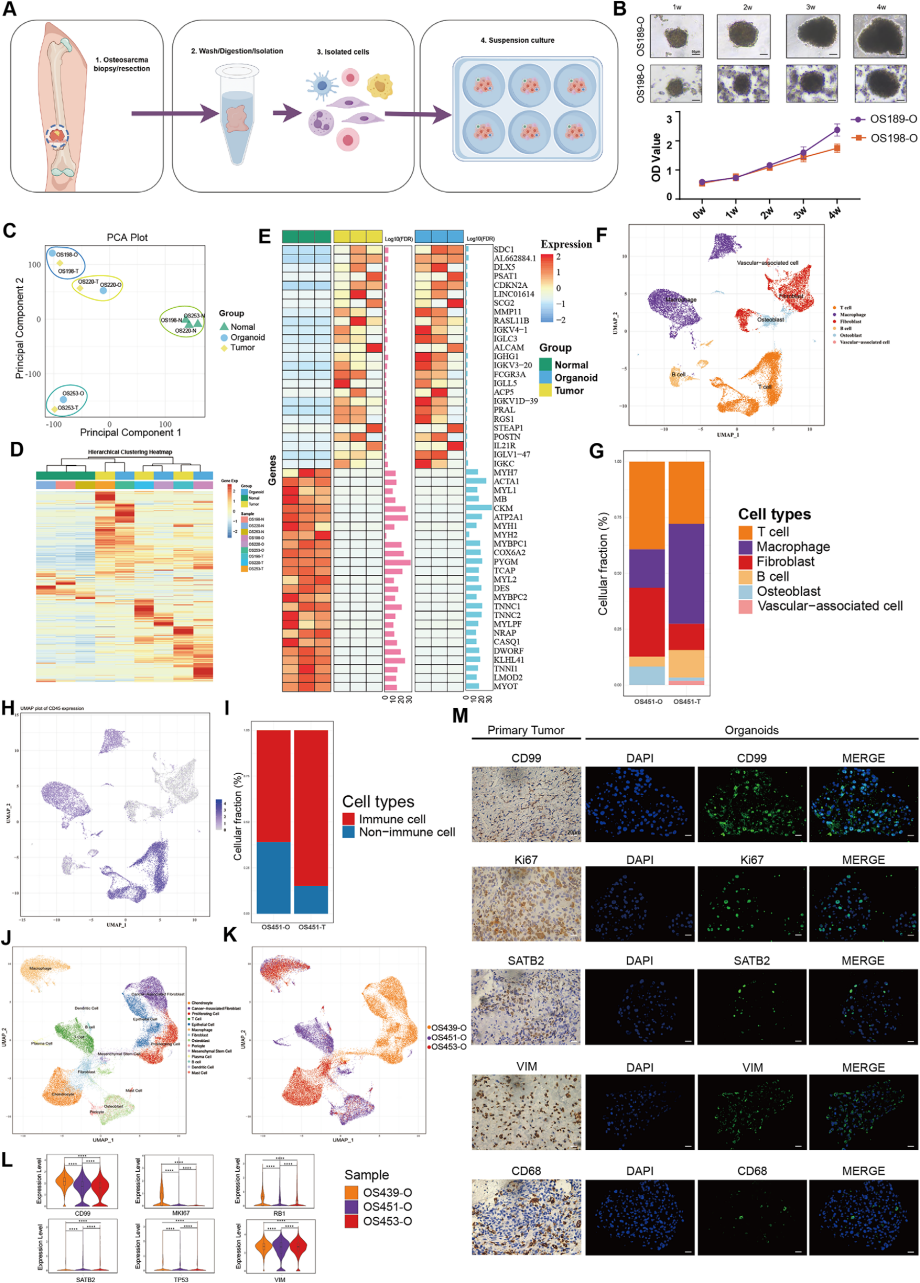
Establishment and Characterization of OSOs. A) Schematic representation of the standardized protocol for constructing OSOs, including tissue collection, enzymatic digestion, and culture. B) Growth curves of two OSOs (OS189‐O and OS198‐O), demonstrating the capacity for sustained growth over time, scale bars, 50 µm. C) PCA of RNA‐sequencing data from three patients, showing distinct clustering of normal, tumor, and OSO samples. D) Hierarchical clustering heatmap of gene expression in normal tissues, tumors, and OSOs. E) Heatmap of the top 25 upregulated and downregulated genes in tumor samples compared to normal tissues, showing that OSOs recapitulate the differential expression patterns of tumors. F,G) UMAP and bar plots comparing the cellular composition of paired tumor and OSO samples at the single‐cell level. H,I). UMAP and bar plots show the presence of CD45+ immune cells in OSOs. J) UMAP plot of pooled OSO samples from three patients, demonstrating heterogeneity in cellular composition across different patients. K) UMAP with patient sample annotation, highlighting patient‐specific differences in cell type distribution. L) Violin plots depict the expression of key genes (CD99, MKI67, RB1, SATB2, TP53, and VIM) across OSOs from three patients. They show variability in gene expression and reflect patient‐specific heterogeneity. M. IHC staining of tumor tissue and IF staining of OSO sections for CD99, VIM, CD68, KI67, and SATB2, showing positive expression in both tumor and OSO tissues, scale bars, 20 µm. ****P < 0.001.

The process of establishing the OSOs is shown in **Figure** **1**A. The growth curves of the two OSOs demonstrated a continuous growth potential (Figure 1B). Principal component analysis (PCA) of RNA sequencing data from normal tissues, tumors, and OSOs derived from three patients revealed distinct clustering patterns. Normal tissues were observed to form a distinct cluster, while tumors and their corresponding organoids constituted a separate cluster. This observation suggested a convergence in gene expression profiles between tumors and their organoids (Figure 1C; Figure S1A, Supporting Information). A heatmap generated from hierarchical clustering analysis provided further evidence supporting the similarity between tumors and organoids, highlighting their distinction from normal tissues (Figure 1D). Gene expression analysis showed that the organoids closely mirrored tumor characteristics. Compared to normal tissues, a heatmap of the top 25 upregulated and 25 downregulated genes in tumors displayed consistent expression patterns in the organoids (Figure 1E).

Single‐cell RNA sequencing (scRNA‐seq) provided insights into the cellular composition of organoids. We performed scRNA‐seq on three organoids and one in situ tumor. Light microscopy images of the organoids were shown in Figure S1B (Supporting Information). Paired single‐cell analyses of the in situ tumor and its corresponding organoids (OS451‐T and OS451‐O), after batch correction and cell annotation (Figure S1D,E, Supporting Information), revealed through UMAP and bar plot analyses that, except for vascular‐related cells, all other cell types were present in the organoids (Figure 1F,G). Additionally, UMAP and bar plot analyses of *CD45*+ cells confirmed the presence of immune cells within the organoids (Figure 1H,I). The combined UMAP plot of organoid samples from three patients (OS439‐O, OS451‐O, and OS453‐O) highlighted the diverse cellular composition (Figure 1J; Figure S1F,G, Supporting Information). Annotation of the UMAP plot using sample information revealed significant inter‐patient differences in cell type distribution (Figure 1K). Violin plots of *CD99*, *MKI67*, *RB1*, *SATB2*, *TP53*, and *VIM* expression levels across the organoids from three patients demonstrated variable gene expression patterns, reflecting patient‐specific heterogeneity (Figure 1L). Immunohistochemistry (IHC) staining of patient tumor sections and immunofluorescence (IF) staining of organoid sections for CD99, VIM, CD68, KI67, and SATB2 showed positive expression in both, confirming histopathological similarities (Figure 1M; Figure S2A, Supporting Information). Additionally, the organoids demonstrated stem cell characteristics and tumorigenic potential, as confirmed by the mouse renal capsule tumor formation experiment and analysis of tumor sections (Figure S2B, Supporting Information).

### 2.2 ERCC6 Identification through Cisplatin‐Resistant Organoids and CRISPR Screening

**Figure 2:**
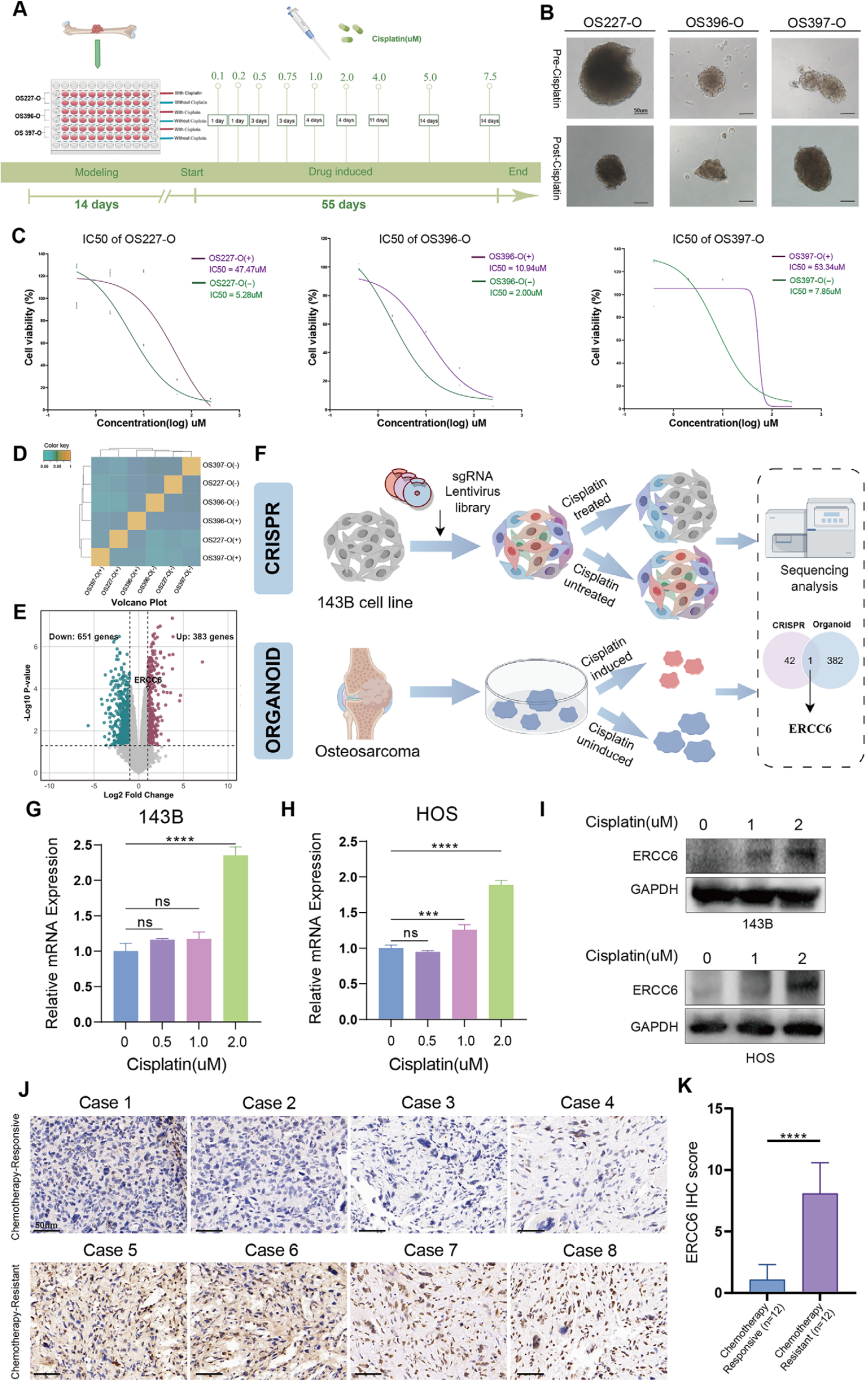
Identification of ERCC6 as a Cisplatin Resistance Gene. A) Time course of cisplatin treatment to induce resistance in three OSOs (OS395‐O, OS396‐O, OS403‐O). (Created by FigDraw, ID: UOURT03073). B) Representative bright‐field images comparing cisplatin‐resistant and non‐resistant OSOs. Scale bars, 50 µm. C) IC50 comparison between cisplatin‐resistant and non‐resistant OSOs for three patient‐derived organoids (OS395‐O, OS396‐O, OS403‐O), showing a significant increase in IC50 in resistant OSOs. (+), with cisplatin; (−), without cisplatin. D) Correlation heatmap of gene expression in resistant and non‐resistant OSOs, showing high intra‐group correlation. (+), with cisplatin; (−), without cisplatin. E) Volcano plot of differentially expressed genes between cisplatin‐resistant and non‐resistant OSOs, highlighting key upregulated and downregulated genes. F) Schematic of CRISPR screen workflow, combining cisplatin‐induced resistance in OSOs with CRISPR/Cas9 screening. (Created by FigDraw, ID: UYWPPcc232). G,H) RT‐qPCR showing increased ERCC6 mRNA expression in cisplatin‐treated 143B and HOS cells. I) WB analysis of ERCC6 protein levels in cisplatin‐treated 143B and HOS cells. J) IHC analysis of ERCC6 expression in chemotherapy‐responsive versus chemotherapy‐resistant patient tumor samples, with representative images from each group. K) IHC scoring reveals that ERCC6 expression is significantly higher in chemotherapy‐resistant patients. Data are presented as the mean ± SD. ns > 0.05, ***P < 0.001, ****P < 0.0001.

We innovatively induced cisplatin resistance in OSOs. **Figure** **2**A showed the timeline for inducing resistance in organoids from three patients (OS227‐O, OS396‐O, and OS397‐O), with one group exposed to cisplatin continuously and the other kept untreated. Light microscopy images (Figure 2B) revealed distinct morphological differences between resistant and non‐resistant organoids. Compared to non‐resistant organoids, the resistant organoids exhibited a more compact spheroid structure with smoother edges. These morphological changes may reflect underlying cellular adaptations associated with the development of drug resistance. The half maximal inhibitory concentration (IC50) values for cisplatin were significantly higher in resistant organoids compared to non‐resistant ones, as shown in Figure 2C (IC50 values: OS227‐O, 5.28 µm vs 47.47 µm; OS396‐O, 2.00 µm vs 10.94 µm; OS397‐O, 7.85 µm vs 53.34 µm). RNA sequencing data revealed distinct gene expression profiles, with a heatmap (Figure 2D) demonstrating high correlation within resistant and non‐resistant groups, respectively. Differential gene expression analysis identified significant upregulation and downregulation of genes in resistant organoids (Figure 2E). KEGG analysis revealed significant enrichment of differentially expressed genes in the phagosome and cell adhesion molecules pathways. Upon further analysis of the Environmental Information Processing section, it was discovered that the MAPK and PI3K/AKT pathways exhibited significant enrichment (Figure S2C,D, Supporting Information). In addition, we performed a whole genome‐wide CRISPR screen under cisplatin treatment. Pathway enrichment analysis of the differentially enriched genes revealed significant enrichment in “platinum drug resistance” and “nucleotide excision repair” pathways. Furthermore, separate enrichment analyses were conducted for both upregulation and downregulation selected genes (Figure S3A, Supporting Information). These results further support the involvement of DNA repair mechanisms in cisplatin resistance. By integrating these data with CRISPR screening results (Figure S3B, Supporting Information), we identified ERCC6 as the sole gene upregulated in resistant organoids and positively selected in CRISPR screens (Figure 2F). Reverse transciption quantitative PCR (RT‐qPCR) (Figure 2G,H) and western blot (WB) analysis (Figure 2I) confirmed that ERCC6 mRNA and protein levels were significantly elevated in 143B and HOS cells under cisplatin pressure. Additionally, IHC analysis was conducted on samples from 12 chemotherapy‐responsive patients and 12 chemotherapy‐resistant patients. Figure 2J displays representative IHC images from four patients in each group, highlighting differences in ERCC6 expression. IHC scoring results (Figure 2K) revealed that ERCC6 expression was significantly higher in chemotherapy‐resistant patients compared to chemotherapy‐responsive patients, suggesting a correlation between ERCC6 expression and chemotherapy resistance.

### 2.3 Functional Validation of ERCC6 in Cisplatin Sensitivity

**Figure 3:**
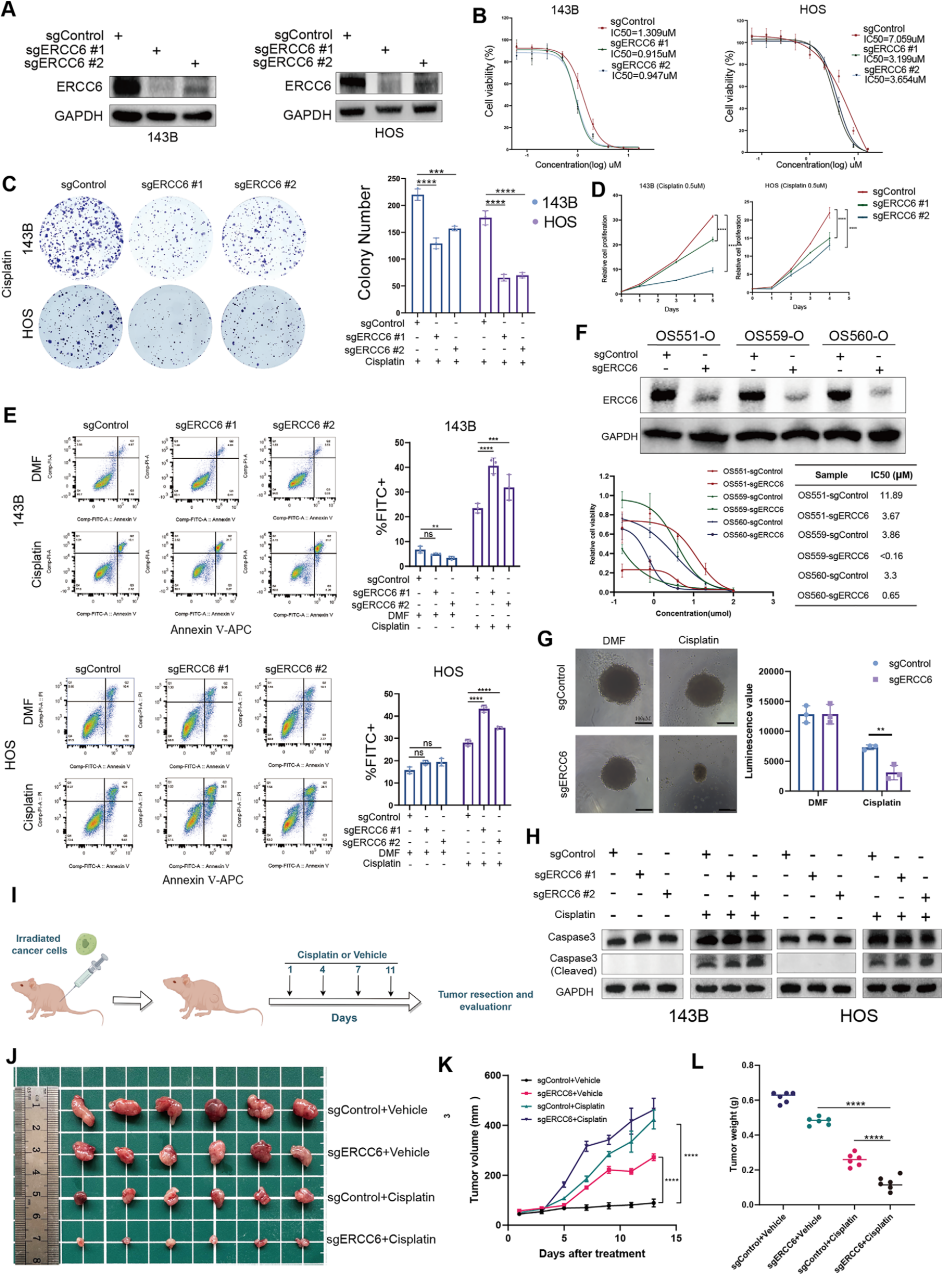
Functional Validation of ERCC6 in Cisplatin Sensitivity. A) WB confirming ERCC6 knockdown in 143B and HOS cell lines using two sgRNAs. B) IC50 values showing increased cisplatin sensitivity in ERCC6 knockdown cells compared to control cells. C) Clonogenic assay results showing reduced colony formation in ERCC6 knockdown cells under cisplatin treatment. (0.5 µm Cisplatin for 143B and HOS cells). D) CCK8 demonstrating decreased proliferation rates in ERCC6 knockdown cells when exposed to cisplatin. E) Flow cytometry analysis showing increased apoptosis rates in ERCC6 knockdown cells treated with cisplatin, as indicated by Annexin V staining. (2 µm Cisplatin for 143B and HOS cells) F) Following sgERCC6 (#1) knockdown, WB results and IC50 detection were conducted on three patient‐derived organoids (OS551‐O, OS559‐O, and OS560‐O). G) Representative bright‐field images and cell viability assays of patient‐derived organoids (OS551‐O, OS559‐O, and OS560‐O) following sgERCC6 (#1) knockdown under 5 µm cisplatin treatment. H) Western blot analysis of caspase‐3 and cleaved caspase‐3, showing increased levels of cleaved caspase‐3 in ERCC6 knockdown cells under cisplatin treatment. I) Experimental design of an OS model under ERCC6 knockdown conditions (Cisplatin, 2 mg kg^−1^, once every three days, intraperitoneally). (Created by FigDraw, ID: IITWPf1f3f). J) Tumor images. K) Tumor growth curves showing that ERCC6 knockdown significantly inhibited tumor growth in the presence of cisplatin. L) Final tumor weights from the xenograft model, confirming the inhibitory effect of ERCC6 knockdown on tumor growth under cisplatin treatment. Data are presented as the mean ± SD. ns > 0.05, **P < 0.01, ***P < 0.001, and ****P < 0.0001.

WB analysis confirmed the successful knockdown of ERCC6 in both 143B and HOS cell lines (**Figure** **3**A). ERCC6 knockdown significantly reduced the IC50 values for cisplatin in both cell lines, indicating increased sensitivity (Figure 3B). Clonogenic assays showed a marked reduction in colony formation under cisplatin treatment in ERCC6 knockdown cells compared to controls (Figure 3C; Figure S3C, Supporting Information). Although ERCC6‐deficient cells showed significantly decreased proliferation in both conditions, the magnitude of inhibition was greater under cisplatin treatment (Figure 3D) compared to cisplatin‐free conditions (Figure S3D, Supporting Information). Flow cytometry analysis demonstrated higher apoptosis rates in ERCC6 knockdown cells treated with cisplatin (Figure 3E). Subsequently, we collected three cases of OSOs (OS551‐O, OS559‐O, OS560‐O) and successfully knocked down ERCC6 using sgERCC6#1 transfection, as evidenced by WB analysis. The IC50 results indicated a significant increase in cisplatin sensitivity after knockout (Figure 3F). In addition, organoids derived from the three patients were treated with 5 µM cisplatin, and those in the ERCC6 knockdown group exhibited pronounced shrinkage and disintegration, along with substantially reduced viability relative to controls (Figure 3G). WB analysis for caspase‐3 and cleaved caspase‐3 showed that cleaved caspase‐3 was barely detectable in all groups without cisplatin. However, under cisplatin treatment, ERCC6 knockdown cells exhibited significantly higher levels of cleaved caspase‐3, indicating enhanced apoptosis (Figure 3H). In vivo experiments showed that ERCC6 knockdown significantly inhibited tumor growth in the presence of cisplatin (Figure 3I,J). Growth curves (Figure 3K) and final tumor weights (Figure 3L) all confirmed this inhibitory effect. We conducted Ki67 and cleaved caspase‐3 IHC on sections of mouse tumors, revealing that after knocking out ERCC6, proliferation was significantly reduced, and apoptosis was significantly increased under the influence of cisplatin (Figure S3E,F, Supporting Information). These results demonstrated that ERCC6 knockdown enhances cisplatin sensitivity in both in vitro and in vivo OS cells.

### 2.4 ERCC6's Relationship with Downstream AKT and Identification of Binding Partner HNRNPM

**Figure 4:**
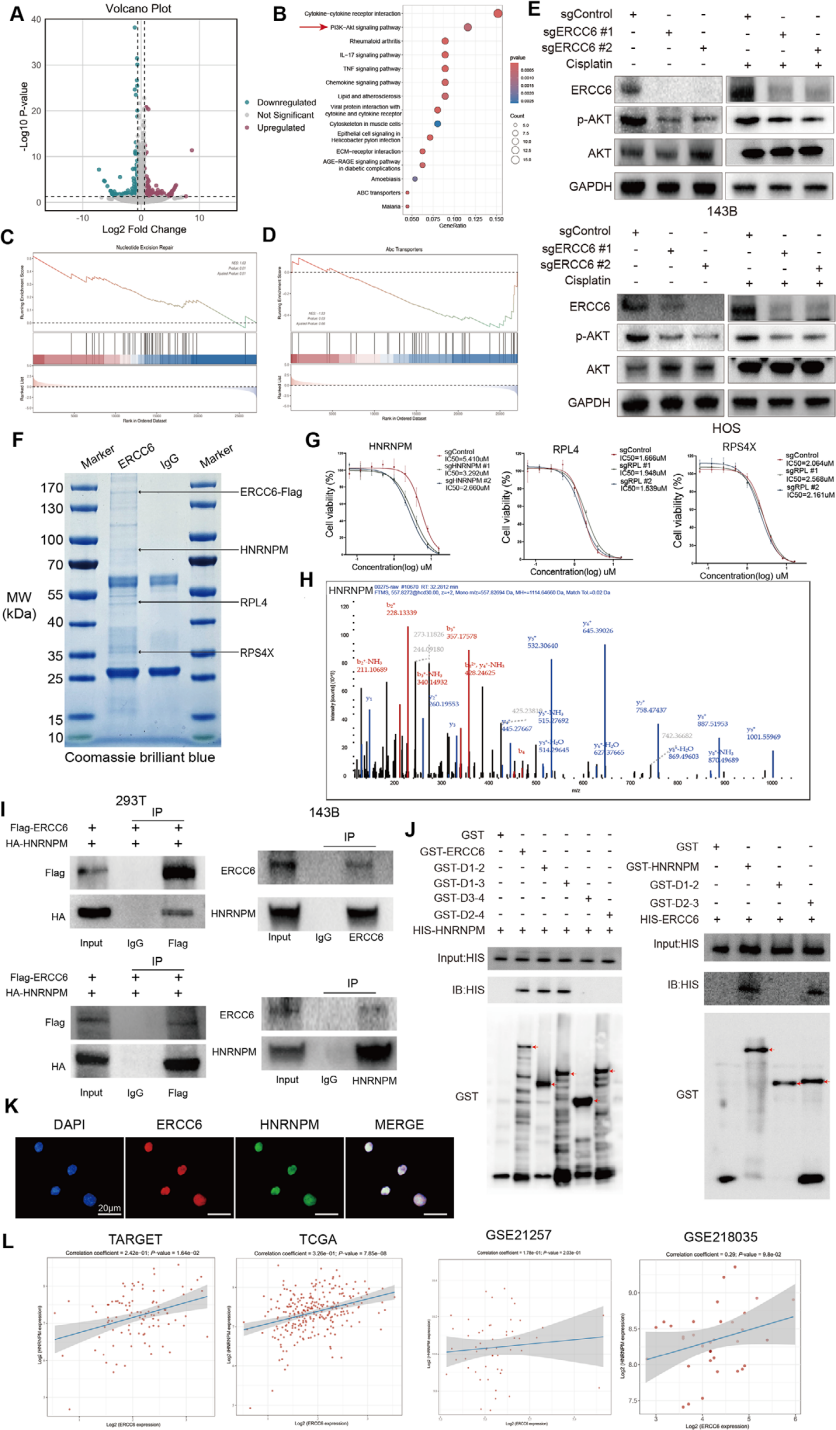
ERCC6 Regulation of AKT Pathway and Identification of Binding Partner HNRNPM. A) Volcano plot of differentially expressed genes from RNA‐seq analysis of ERCC6 knockout 143B cells. B) KEGG pathway analysis shows significant enrichment of the PI3K/AKT pathway in ERCC6 knockout cells. C,D) Gene set enrichment analysis showing reduced expression of “Nucleotide Excision Repair” and “ABC transporters” pathways in ERCC6 knockout cells. E) WB analysis of AKT and p‐AKT, showing reduced p‐AKT levels in ERCC6 knockdown 143B and HOS cells. F) Coomassie blue staining of Co‐IP experiment identifying ERCC6 binding partners, including HNRNPM. G) The IC50 value of cisplatin for potential co‐acting proteins in 143B cells. H) Mass spectrometry confirming HNRNPM as an ERCC6 binding partner. I) Co‐IP experiments in 293T and 143B cells confirming ERCC6‐HNRNPM interaction. J) GST pull‐down assay identifying the interaction domains between ERCC6 and HNRNPM. K) IF co‐localization of ERCC6 and HNRNPM in 143B cells, scale bars, 20 µm. L) Positive correlation between ERCC6 and HNRNPM expression, as shown by analysis of multiple public datasets (TARGET, TCGA, GSE21257, and GSE218035).

Differential gene expression analysis between ERCC6 knockout and control cells revealed significant changes (**Figure** **4**A; Figure S4A, Supporting Information). KEGG pathway analysis indicated enrichment in the PI3K/AKT pathway (Figure 4B; Figure S4B, Supporting Information), with reduced enrichment in the “Nucleotide Excision Repair” and “ABC transporters” pathways (Figure 4C,D). The PI3K/AKT pathway was also significantly enriched in previous organoid differential analyses. WB analysis confirmed that ERCC6 knockout led to a marked reduction in p‐AKT levels under both cisplatin‐free and cisplatin‐treated conditions (Figure 4E). Further analysis showed that after knocking down ERCC6, the splicing pathway was significantly reduced (Figure S4C, Supporting Information). Co‐immunoprecipitation (CO‐IP) followed by mass spectrometry identified several potential binding partners of ERCC6 (Figure 4F, Figure S4D and Table S4, Supporting Information). Among the top 25 candidates ranked by abundance, we excluded keratins and housekeeping proteins, and further selected those showing distinct differential bands in the IP gels. Based on these criteria and supporting literature, HNRNPM,^[^ ^15^, ^16^, ^17^ ^]^ RPL4,^[^ ^18^, ^19^ ^]^ and RPS4X^[^ ^20^, ^21^ ^]^ were prioritized for further validation due to their reported involvement in tumor progression or chemoresistance. IC50 assays revealed that only HNRNPM knockdown significantly reduced cisplatin resistance in 143B cells (Figure 4G; Figure S4E, Supporting Information). Mass spectrometry confirmed HNRNPM in the ERCC6 CO‐IP complex (Figure 4H). Further validation through CO‐IP in 293T cells and 143B cells confirmed the ERCC6‐HNRNPM interaction (Figure 4I). GST pull‐down assays identified the nucleotide‐binding domain (NBD, D1 amino acids 1–510) of ERCC6 as the critical region mediating its interaction with HNRNPM (Figure 4J). Conversely, ERCC6 was found to interact with HNRNPM through its D3 region spanning amino acids 653–729 (Figure 4J). Functional rescue experiments further validated the significance of this interaction: while full‐length ERCC6 effectively increase the cisplatin IC50 in osteosarcoma cells, the NBD‐truncated mutant failed to do so. Similarly, deletion of amino acids 653–729 in HNRNPM resulted in no increase in cisplatin IC50 compared to the full‐length protein (Figure S4F, Supporting Information). To gain deeper insights into the interaction interface, molecular docking analysis was performed (Figure S4G, Supporting Information), which predicted key contact residues at the binding site. Specifically, GLU130 and TYR133 of ERCC6 were identified as potential interaction residues, while RPO660, ASP662, and THR664 of HNRNPM were predicted to form complementary contacts. These findings support the biochemical and functional data, providing structural insights into the ERCC6–HNRNPM interaction and its role in mediating chemoresistance in osteosarcoma.

Immunofluorescence staining showed colocalization of ERCC6 and HNRNPM in 143B cells (Figure 4K). The correlation between ERCC6 and HNRNPM was analyzed using four public databases (including osteosarcoma in TARGET, sarcoma in TCGA, GSE21257, and GSE218035), and the results showed a positive correlation (Figure 4L). Similarly, IHC analysis of HNRNPM in 12 pairs of chemotherapy‐resistant and ‐sensitive patient samples revealed significantly higher HNRNPM expression in the chemotherapy‐resistant group (Figure S4H, Supporting Information).

### 2.5 HNRNPM Plays a Crucial Role in Modulating the Sensitivity of OS to Cisplatin

**Figure 5:**
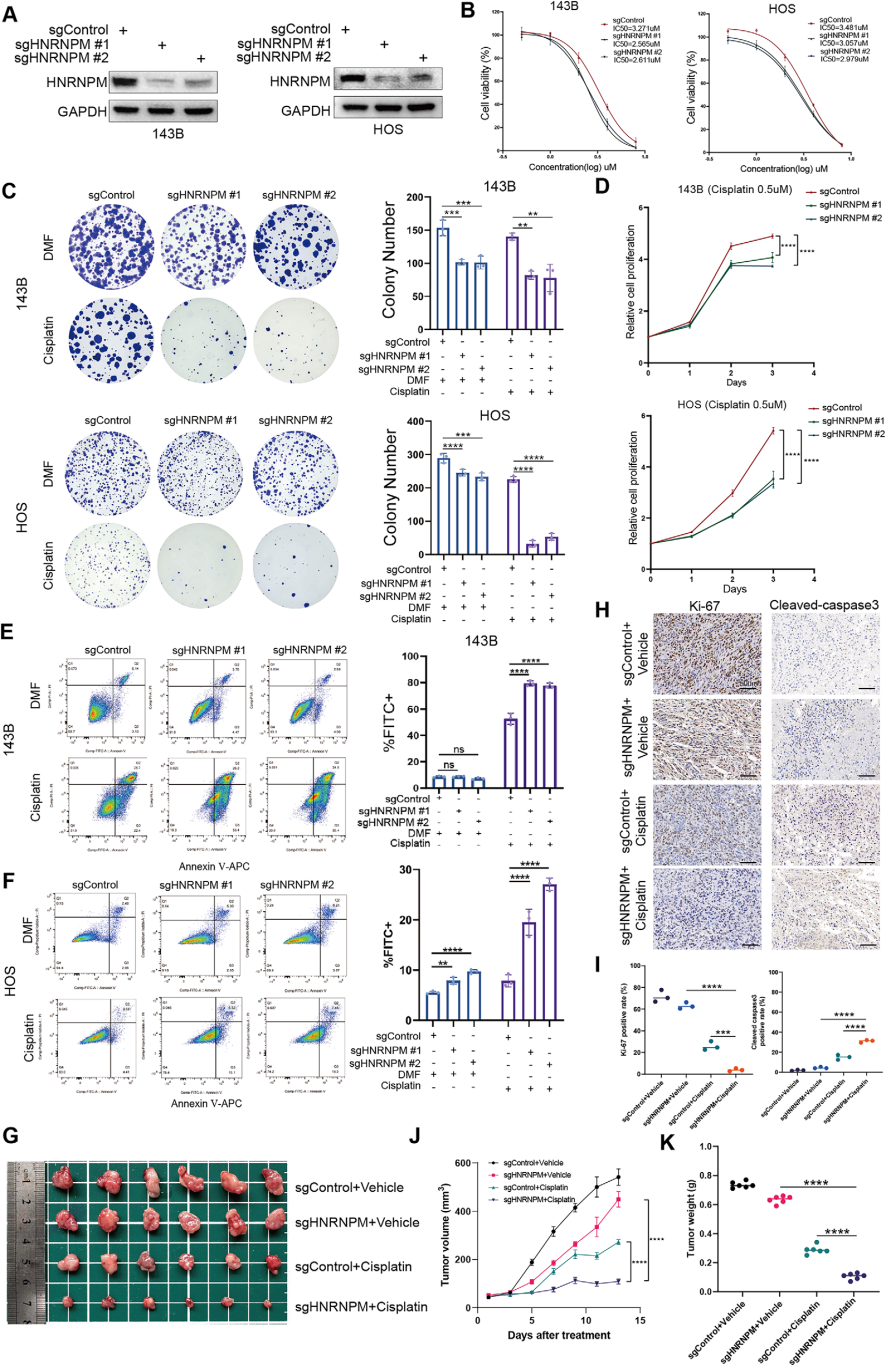
Functional Validation of HNRNPM in Cisplatin Sensitivity. A) WB confirming HNRNPM knockdown in 143B and HOS cell lines using sgRNAs. B) IC50 values showing increased cisplatin sensitivity in HNRNPM knockdown cells. C) Clonogenic assay results showing reduced colony formation in HNRNPM knockdown cells under cisplatin treatment. D) CCK8 demonstrating decreased cell proliferation in HNRNPM knockdown cells under cisplatin treatment. E,F) Flow cytometry analysis showing increased apoptosis rates in HNRNPM knockdown cells treated with cisplatin. (2 µm Cisplatin for 143B and HOS cells). G) Tumor images from the xenograft assay in mice. H,I) IHC staining of mouse tumor sections showing decreased Ki‐67 and increased cleaved caspase‐3 expression in HNRNPM knockdown tumors treated with cisplatin, scale bars, 50 µm. J) Tumor growth curves from the xenograft model showing reduced tumor growth in HNRNPM knockdown tumors treated with cisplatin. K) Final tumor weights, confirming the tumor‐inhibitory effect of HNRNPM knockdown under cisplatin treatment. Data are presented as the mean ± SD. ns > 0.05, **P < 0.01, ***P < 0.001, and ****P < 0.0001.

**Figure 6:**
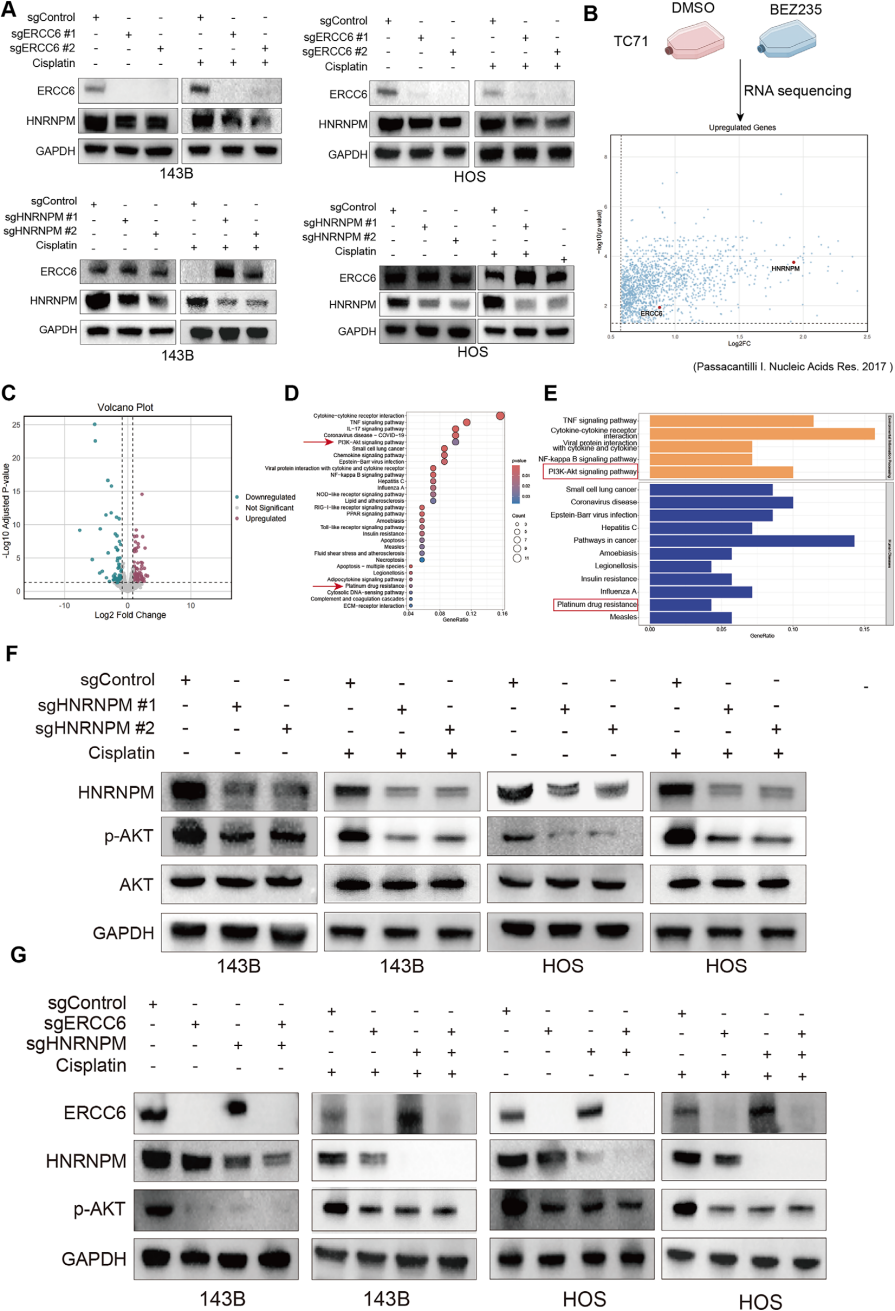
Interaction between ERCC6 and HNRNPM in Modulating PI3K/AKT Pathway. A) WB showing that ERCC6 knockdown reduces HNRNPM expression under cisplatin treatment, while HNRNPM knockdown increases ERCC6 expression. B) Reanalysis of data from Passacantilli et al. shows that PI3K inhibitor BEZ235 elevates ERCC6 and HNRNPM levels. C) Volcano plot of differentially expressed genes from RNA‐seq analysis of HNRNPM knockdown cells, highlighting significant changes. D,E) KEGG pathway analysis showing enrichment in the PI3K/AKT and cisplatin resistance pathways in HNRNPM knockdown cells. F) Western blot showing reduced p‐AKT levels in HNRNPM knockdown cells, with unchanged total AKT levels. G) p‐AKT levels in ERCC6 knockdown, HNRNPM knockdown, and double knockdown cells, with no significant difference between single and double knockdowns.

WB confirmed successful HNRNPM knockdown in 143B and HOS cell lines using sgRNAs (**Figure** **5**A). IC50 assays showed that HNRNPM knockdown significantly increased cisplatin sensitivity in both cell lines (Figure 5B). Clonogenic assays revealed reduced colony formation under cisplatin treatment in HNRNPM knockdown cells (Figure 5C). Proliferation assays indicated decreased cell proliferation rates in HNRNPM knockdown cells under cisplatin pressure (Figure 5D). Flow cytometry analysis demonstrated higher apoptosis rates in HNRNPM knockdown cells treated with cisplatin (Figure 5E,F). In vivo, HNRNPM knockdown significantly inhibited tumor growth in the presence of cisplatin, as demonstrated by representative tumor images (Figure 5G), growth curves (Figure 5J), and final tumor weights (Figure 5K). IHC staining of mouse tumor sections revealed decreased Ki‐67 and increased cleaved caspase‐3 expression in HNRNPM knockdown tumors treated with cisplatin (**Figure** **6**H,I). These results confirmed that HNRNPM knockdown enhanced cisplatin sensitivity in OS cells both in vitro and in vivo.

### 2.6 ERCC6 and HNRNPM Interaction and Their Effects on the PI3K/AKT Pathway

We examined HNRNPM and ERCC6 expression in 143B and HOS cells under cisplatin treatment. ERCC6 knockdown reduced HNRNPM expression, with a more significant reduction under cisplatin. HNRNPM knockdown did not affect ERCC6 expression without cisplatin but increased ERCC6 expression under cisplatin (Figure 6A). Reanalysis of data from Passacantilli I et al.^[^ ^22^ ^]^ showed that PI3K inhibitor BEZ235 treatment elevated ERCC6 and HNRNPM levels in the Ewing sarcoma TC71 cell line (Figure 6B). RNA sequencing of HNRNPM knockdown cells revealed significant differential gene expression (Figure 6C). KEGG analysis indicated enrichment in the PI3K/AKT and cisplatin resistance pathways (Figures 6D,E). WB showed unchanged AKT levels but significantly reduced p‐AKT in HNRNPM knockdown cells under both cisplatin‐free and cisplatin‐treated conditions (Figure 6F). p‐AKT levels were also reduced in ERCC6 knockdown, HNRNPM knockdown, and ERCC6/HNRNPM double knockdown cells, with no significant difference between single and double knockdowns (Figure 6G). These results suggest that ERCC6 and HNRNPM modulate the PI3K/AKT pathway, impacting cisplatin resistance.

### 2.7 Alternative Splicing Modulation by ERCC6 and HNRNPM

**Figure 7:**
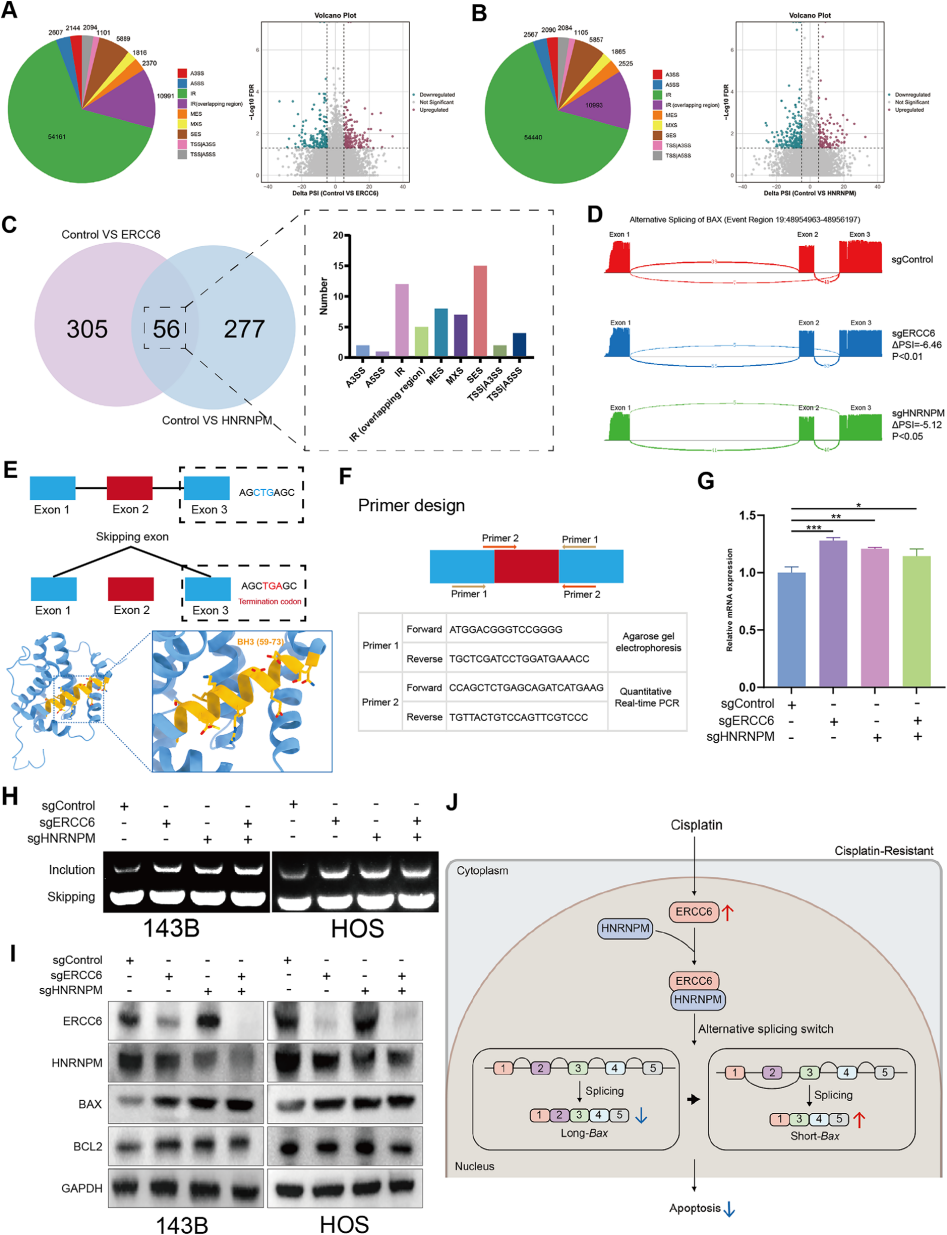
Alternative Splicing Modulation by ERCC6 and HNRNPM. A) Pie chart and volcano plot showing significant alternative splicing events following ERCC6 knockdown. B) Pie chart and volcano plot showing alternative splicing events following HNRNPM knockdown, highlighting the role of BAX. C) Bar chart categorizing 56 common alternative splicing events between ERCC6 and HNRNPM knockdowns. D) IGV visualization of BAX exon 2 skipping across control and knockdown groups. E) Schematic illustrating two BAX splicing variants: full‐length BAX and the truncated version resulting from exon 2 skipping, leading to a premature stop codon. F) Primer designs for exons 1 and 3 (for agarose gel electrophoresis) and exons 2 and 3 (for qRT‐PCR). G) qRT‐PCR results showing increased full‐length BAX expression in knockdown groups compared to controls. H) Agarose gel electrophoresis showing full‐length and skipped BAX bands in all four groups, with increased full‐length BAX in knockdown groups. I) Western blot showing increased BAX protein levels in knockdown groups, with no significant change in BCL2 expression. J) Mechanistic diagram summarizing the role of ERCC6 and HNRNPM in modulating BAX splicing and the PI3K/AKT pathway, influencing cisplatin resistance. Data are presented as the mean ± SD. *P < 0.05, **P < 0.01, and ***P < 0.001.

ERCC6 knockdown resulted in significant alternative splicing changes, as shown by a classification pie chart and a volcano plot comparing differential splicing events with the control (**Figure** **7**A). KEGG and GO analyses of differentially expressed genes revealed that BAX plays a pivotal role in multiple pathways (Figure S5A,B, Supporting Information). WB further confirmed an increase in BAX expression upon knockdown of ERCC6 (Figure S5C). Similarly, HNRNPM knockdown led to distinct splicing changes, illustrated by a pie chart and a volcano plot (Figure 7B). According to the analysis of the alternative splicing pathway of HNRNPM (Figure S5D,E, Supporting Information), BAX also played an important role, and WB showed that BAX expression increased when HNRNPM was knocked down (Figure S5F). Overlapping splicing events between ERCC6 and HNRNPM knockdowns identified 56 common events, represented in a bar chart categorizing the types of splicing events (Figure 7C). KEGG enrichment analysis of the common differentially spliced genes highlighted BAX as a key player (Figure S5G, Supporting Information). The specific splicing event involves the skipping of exon 2 in BAX (target region: 19:48955687‐48955833). IGV visualization confirmed exon 2 skipping in BAX across all three groups (Figure 7D). The schematic in Figure 7E illustrated two splicing variants: the full‐length BAX (L‐BAX) and a truncated version resulting from exon 2 skipping (S‐BAX). This splicing event alters the translation reading frame, leading to the introduction of a premature stop codon (TGA) in exon3 within the BH3 domain.

Primers designed for exons 1 and 3 (for agarose gel electrophoresis) and for exons 2 and 3 (for qRT‐PCR) are shown in Figure 7F. The results indicated a significant increase in L‐BAX expression in ERCC6 knockdown, HNRNPM knockdown, and double knockdown groups compared to the control (Figure 7G). Agarose gel electrophoresis confirmed that all four groups exhibited both full‐length and skipped bands, with no significant difference in the skipped bands but an increase in the full‐length bands in the knockdown groups (Figure 7H). WB analysis revealed increased BAX protein levels in the knockdown groups, with no significant change in BCL2 expression (Figure 7I).

Figure 7J presents a mechanistic diagram highlighting the roles of BAX alternative splicing in mediating cisplatin resistance. These results underscore the critical role of ERCC6 and HNRNPM in regulating alternative splicing events, particularly in BAX, contributing to apoptosis and cisplatin sensitivity in OS cells.

## 3 Discussion

Our study highlights the use of patient‐derived OSOs in uncovering the mechanisms of cisplatin resistance, elucidates the critical roles of ERCC6 and HNRNPM in mediating cisplatin resistance in OS, focusing on the PI3K/AKT pathway and alternative splicing of *BAX*. These findings provide significant insights into the molecular mechanisms underlying chemo‐resistance and identify potential therapeutic targets for overcoming drug resistance in OS.

In clinical practice, our primary objective was to search for alternative or combined drugs for OS patients with chemotherapy resistance. Individualized drug screening can offer precise medication strategies for patients, while the efficiency of existing in vitro models is low. Patient‐derived OSOs present an opportunity to investigate and characterize acquired chemoresistance more comprehensively. Compared with organoid models, primary tumors present significant challenges in obtaining matched pre‐ and post‐drug resistance samples and in exploring resistance mechanisms specific to individual chemotherapeutic agents. Additionally, traditional cell lines fail to recapitulate inter‐patient heterogeneity and exhibit limited clinical relevance, further constraining their translational utility. We successfully established OSOs that faithfully recapitulate the original tumor characteristics. These organoids demonstrated consistent growth dynamics, gene expression profiles, and cellular composition with the in situ tumors. In addition to showing high fidelity, scRNA‐seq highlighted the heterogeneity among organoids derived from different patients, emphasizing the model's relevance for personalized medicine.

Long‐term cisplatin induction of OSOs to establish a drug‐resistant model closely simulates the resistance process observed in patients. The induction of resistance in organoids, combined CRISPR screening in 143B cell line, provided a powerful method for pinpointing key resistance driver genes.^[^ ^23^ ^]^ ERCC6 was identified as a pivotal factor in cisplatin resistance through a combination of organoid‐based drug induction and CRISPR screening. ERCC6 plays an essential role in transcription‐coupled nucleotide excision repair, which removes RNA polymerase II‐blocking lesions from active genes’ transcribed strands quickly.^[^ ^24^, ^25^ ^]^ Recent research has shown a correlation between ERCC6 expression levels and cancer prognosis.^[^ ^26^ ^]^ For instance, higher levels of ERCC6 have been associated with poorer outcomes in some cancer types, including gastric cancer,^[^ ^27^ ^]^ lung cancer,^[^ ^28^ ^]^ bladder cancer^[^ ^29^ ^]^ and breast cancer.^[^ ^30^ ^]^ However, the role of ERCC6 in OS and its cisplatin resistance mechanism remained unclear.

We prioritized the use of cell lines for in vitro mechanistic experiments due to their suitability for high‐throughput screening, ease of manipulation, and well‐established experimental reproducibility. To enhance the robustness of our findings, we subsequently utilized the organoid model for validation, ensuring greater biological relevance and translational potential. Our results demonstrated that ERCC6 knockdown significantly increased cisplatin sensitivity, reduced proliferation, promoted apoptosis, and suppressed tumor growth in both in vitro and in vivo osteosarcoma models. Moving forward, further expansion of organoid models, while maintaining consistency, will be essential for gaining deeper insights into drug resistance mechanisms and advancing personalized therapeutic strategies and drug screening applications.

The relationship between ERCC6 and the PI3K/AKT pathway was further elucidated through KEGG analysis and WB. ERCC6 knockdown led to decreased p‐AKT levels, highlighting ERCC6's involvement in activating this critical survival pathway. This aligns with existing literature on the PI3K/AKT pathway's role in promoting cell survival and chemoresistance.^[^ ^31^, ^32^ ^]^ Additionally, HNRNPM was identified as a binding partner of ERCC6, further contributing to the complexity of the resistance network. We mapped the interaction domains between the two proteins and predicted specific binding residues. Functional validation demonstrated that these domains play a critical role in modulating cisplatin sensitivity, thereby providing potential targets for future drug development. HNRNPM knockdown mirrored the effects of ERCC6 knockdown, indicating a co‐regulatory role in the PI3K/AKT pathway. Interestingly, ERCC6 and HNRNPM appeared to influence each other's protein expression, suggesting a potential reciprocal regulatory mechanism. This observation highlighted a promising direction for future research aimed at further elucidating the functional role of ERCC6 and its involvement in cisplatin resistance.

In addition, HNRNPM is a ubiquitously expressed nuclear RNA‐binding protein and plays a critical role in alternative splicing.^[^ ^33^, ^34^ ^]^ In previous studies, it was reported that HNRNPM regulates the alternative splicing of CD44 by binding to ESRP1.^[^ ^35^ ^]^ Therefore, we further investigated the effect of ERCC6 binding to HNRNPM on downstream alternative splicing. The KEGG enrichment analysis of ERCC6 identified significant enrichment in the spliceosome pathway. Our analysis of alternative splicing revealed that both ERCC6 and HNRNPM knockdowns significantly impact *BAX* splicing, leading to increased expression of the full‐length *BAX* isoform, which promotes apoptosis. As a critical executioner protein in the mitochondrial‐regulated cell death pathway, the pro‐apoptotic protein BAX plays a crucial role in processes such as development, tissue homeostasis, and immune regulation.^[^ ^36^ ^]^ Importantly, we identified a specific splicing event where exon 2 is skipped in *BAX*, causing a frameshift in the downstream reading frame. This frameshift results in introducing a premature stop codon (TGA) within the BH3 domain, a critical region for *BAX*’s pro‐apoptotic function.^[^ ^37^ ^]^ This alteration generates a truncated, non‐functional BAX protein, as the premature stop codon prevents the translation of the entire BH3 domain, which is essential for BAX's interaction with other pro‐apoptotic proteins. The loss of exon 2 and the subsequent truncation of the protein disrupts normal apoptotic signaling, reducing apoptosis and contributing to cisplatin resistance. Our qRT‐PCR and agarose gel electrophoresis confirmed the increased expression of the full‐length *BAX* isoform in knockdown conditions, suggesting that ERCC6 and HNRNPM modulate *BAX* splicing in a way that favors the production of the functional isoform. These findings provide a direct link between splicing regulation and chemoresistance, as the altered splicing of *BAX*, driven by ERCC6 and HNRNPM, shifts the balance toward apoptosis in the presence of cisplatin. This novel mechanism expands our understanding of how alternative splicing contributes to cisplatin resistance and highlights the therapeutic potential of targeting splicing regulators to overcome resistance in osteosarcoma treatment.

In conclusion, our findings highlighted the critical roles of ERCC6 and HNRNPM in mediating cisplatin resistance in OS through the modulation of the PI3K/AKT pathway and alternative splicing of *BAX*. The innovative approach of combining drug‐induced resistance in organoids with CRISPR screening has discovered novel regulatory mechanisms. ERCC6 and HNRNPM represent promising therapeutic targets for overcoming chemoresistance. Future studies should focus on developing specific inhibitors of ERCC6 and HNRNPM, which could be used in combination with existing chemotherapies to enhance treatment efficacy and improve patient outcomes in OS.

## 4 Experimental Section

### Human Tissues

OS and matched normal tissues were obtained from patients undergoing surgery at the Second Xiangya Hospital, Central South University, with approval from the Ethics Committee (Approval NO. 2020–114, and NO. LYEC2024‐K0146) and in accordance with ethical guidelines. For this study included patients with a confirmed OS diagnosis and those receiving standard MAP chemotherapy (methotrexate, doxorubicin, cisplatin). Before and after standard MAP chemotherapy on OS patients, needle biopsy and surgical resection specimens were collected. CT or MRI imaging was used to assess treatment effectiveness according to RECIST 1.1.^[^ ^38^ ^]^ Further analysis was conducted on samples taken from patients with partial response (chemotherapy‐sensitive) and progressive disease (chemotherapy‐resistant). Informed consent for tissue and clinical data usage was obtained from all patients. Collected tissues were immediately processed for experimental applications, including RNA sequencing, scRNA sequencing, organoid establishment, and IHC.

### Establishment and Culture of OSOs

A standardized protocol was established for constructing OSOs: a) Tissue Collection: Surgical or biopsy tumor tissues from patients were collected and cut into small pieces within 3 h using scissors. b) Enzymatic Digestion: The tissue fragments were digested with collagenase I for 1 h in a shaking incubator at 37 °C. c) Termination of Digestion: Digestion was terminated by adding fetal bovine serum (FBS), followed by filtration through a 100 µm filter. d) Culture: The resulting cell suspension was cultured in an optimized OSO medium, detailed in Table S1 (Supporting Information). In suspension, various irregularly shaped tumor‐derived cells undergo a self‐assembly process, transforming into smooth‐surfaced tumor organoids. e) Passaging: The OSO spheroids were digested into single cells using TrypLE, with a passaging ratio of 1:3. OSOs at passages 4–6 were mixed with Matrigel and injected sub‐renally into one side of the kidney capsule of 5‐week‐old female NOD‐SCID mice. After four weeks, the NOD‐SCID mice were euthanized, and both kidneys were dissected. The tissues were embedded, sectioned, and examined microscopically to assess tumor formation by the organoids in vivo.

### RNA‐seq Analysis of Normal Tissues, Primary Tumors, and Derived OSOs

RNA was extracted from normal tissues, in situ tumors, and OSOs using TRIzol (Thermo Fisher Scientific). Sequencing was performed on the Illumina HiSeq platform using paired‐end sequencing mode. In order to reduce low‐quality scores associated with the ends of Illumina sequencing data, Skewer software was used to dynamically trim adapter sequences and low‐quality fragments. Preprocessed data were then subjected to quality control analysis using FastQC software. As a part of the preprocessing of the sequences, STAR software was used to align them with the human reference genome (hg38). The DESeq2 package was used to identify differentially expressed genes based on *P* values 0.05 and fold changes > 2 based on differential gene expression.^[^ ^39^ ^]^

### Single‐Cell RNA Sequencing

One in situ tumor (OS451‐T) and three OSOs (OS439‐O, OS443‐O, OS451‐O) were collected for single‐cell RNA sequencing. Sequencing and analysis were conducted by Singleron Biotechnologies. Tissue samples were dissociated into single‐cell suspensions using sCelLiVE tissue dissociation solution. The cells were diluted to a concentration of 2.5–3.5×10^5 cells mL^−1^. Single‐cell suspensions were loaded into SCOPE‐chip microfluidic chips, which isolated individual cells based on Poisson distribution. Each cell was captured in a micro‐well along with a unique barcoded bead. Upon cell lysis, mRNA was captured by these beads. Captured mRNA was reverse‐transcribed into cDNA and amplified. The cDNA was fragmented, and sequencing libraries were constructed with adapters suitable for the Illumina sequencing platform. Filtering steps for high‐quality single cells included retaining genes expressed in at least three cells and cells expressing a minimum of 250 genes. Subsequently, more stringent filtering was applied by creating Seurat objects containing only cells with 500 to 6000 expressed genes. To minimize potential biases, cells with mitochondrial gene content exceeding 20%, ribosomal gene content above 30% were also excluded, effectively removing doublets and low‐quality cells. Following quality control of scRNA‐seq data, gene expression profiles were normalized using Seurat's LogNormalize method within the NormalizeData function. Subsequently, highly variable genes were identified using the default parameters of Seurat's FindVariableFeatures function. Batch effects and sample integration were addressed using the Harmony function. Marker genes were used to annotate cells. The TOP 10 markers for each cell type are listed in Table S2 (Supporting Information). scRNA sequencing data was analyzed with Seurat V5.^[^ ^40^ ^]^

### IHC

Following established protocols, IHC staining was performed on paraffin‐embedded sections from human and mouse samples.^[^ ^41^ ^]^ Before embedding the organoids in paraffin, they were wrapped in agarose to ensure their aggregated nature and facilitate sectioning. Clinical samples were stained for ERCC6 (#ab96089, Abcam), while mouse samples were stained for Ki‐67 (#ab16667, Abcam) and cleaved caspase‐3 (#9661, Cell Signaling Technology). Two senior pathologists in dependently evaluated IHC scores for clinical samples using a previously described scoring method. A semi‐quantitative evaluation of staining results was performed by multiplying staining intensity scores with cell positivity scores. Based on the percentage of positive cells, 0 was scored for <5%, 1 for 6%–25%, 2 for 26%–50%, 3 for 51%–75%, and 4 for >75%. Staining intensity was graded on a scale of 0 to 3: 0 (negative), 1 (weak), 2 (moderate), and 3 (strong).^[^ ^42^ ^]^

### RT‐qPCR

Following the manufacturer's instructions, total RNA was extracted from cultured cells using TRIzol (Thermo Fisher Scientific). cDNA was synthesized from 1 µg of total RNA using the Hifair III Reverse Transcriptase (Yeasen Biotechnology Co., Ltd., China). RT‐qPCR was performed using the SYBR Green Master Mix (#AG11746, Accurate Biology, China) on QuantStudio5 (Applied Biosystems, USA). The specific primers used for RT‐qPCR are listed in Table S3 (Supporting Information). Target gene expression levels were normalized to GAPDH using the 2^‐ΔΔCt method.

### WB

The proteins were extracted from tissue samples and cultured cells using RIPA lysis buffer (#P0013, Beyotime) containing protease inhibitors and phosphatase inhibitors. The BCA Protein Assay Kit (Sigma‐Aldrich) was used to determine the protein concentration. The same amount of protein (20–40 µg) was separated separated by SDS‐PAGE on polyacrylamide gel and transferred onto PVDF membranes (Millipore). Blocking with 5% non‐fat milk in TBST (Tris‐buffered saline with 0.1% Tween‐20) was carried out for 1 h at room temperature, followed by incubation with primary antibodies overnight at 4 °C. The following primary antibodies: anti‐ERCC6 (#sc‐398022, Santa, 1:500 dilution), anti‐HNRNPM (Abcam Cat# ab177957, 1:2000 dilution), anti‐Caspase 3/p17/p19 (Proteintech Cat# 19677‐1‐AP, 1:2000 dilution), and anti‐phospho‐AKT (Cell Signaling Technology Cat# 13 038, 1:1000 dilution), anti‐AKT (Proteintech Cat# 60203‐2‐Ig, 1:2000 dilution), BAX (Proteintech Cat# 50599‐2‐Ig, 1:1000 dilution), BCL2 (#68103‐1‐Ig, Proteintech, 1:1000 dilution), GAPDH (#60004‐1‐Ig, Proteintech, 1:5000 dilution) were used. TBST was used to wash the membranes, followed by an hour of room temperature incubation with secondary antibodies (HRP conjugated goat‐anti‐mouse antibody (Zen Bio Cat# 511 103, 1:5000 dilution), and HRP conjugated goat‐anti‐rabbit antibody (Zen Bio Cat# 511 203, 1:5000 dilution). Enhanced chemiluminescence (ECL) was used to visualize the protein bands, and images were captured using the Bio‐Rad Image Lab.

### Establishment of Cisplatin‐Resistant Organoids

To establish cisplatin‐resistant organoids, organoids derived from three patients (OS227‐O, OS395‐O, and OS396‐O) were subjected to continuous cisplatin exposure over 55 days. The concentration of cisplatin was gradually increased from 0 to 7.5 µm. The process was as follows: a) Initial Culturing: Established organoids were initially cultured in an optimized OSO medium without cisplatin. b) Cisplatin Exposure: Starting with a concentration of 0.5 µm, cisplatin was added to the culture medium. The concentration was gradually increased every 5–7 days. c) Final Concentration: After 55 days, the organoids were exposed to a final cisplatin concentration of 7.5 µm. d) Monitoring: The morphology and viability of the organoids were regularly monitored using light microscopy. e) The viability of the cisplatin‐resistant organoids was assessed using the CellTiter‐Glo 3D Cell Viability Assay kit (Promega). Fluorescence was measured, and the data were normalized to organoid volume to calculate the IC50 value for cisplatin.

### RNA Sequencing of Cisplatin‐Resistant and Non‐Resistant OSOs

RNA sequencing of cisplatin‐resistant OSOs and non‐resistant OSOs was performed using the KC‐digitalRNA sequencing method with UMI (Unique Molecular Identifier) deduplication, provided by Wuhan Kangce Technology Co., Ltd, which could accurately sequence minute amounts of RNA.^[^ ^43^ ^]^ Low‐quality reads and adapter sequences were removed from the sequencing data, and the clean reads were aligned to the human reference genome (hg38) using STAR. UMI counts were used to quantify gene expression levels accurately. Differential expression analysis between cisplatin‐resistant and non‐resistant organoids was performed using DESeq2, identifying significantly differentially expressed mRNAs based on P values < 0.05 and a fold change > 1.5.

### CRISPR/Cas9 Knockout Library Screen for Identifying Cisplatin‐Resistant Genes

A genome‐wide CRISPR/Cas9 knockout library screen was performed to identify genes associated with cisplatin resistance in 143B cells (OS cell line). The human GeCKO v2 CRISPR knockout library, which targets ≈19000 genes with 123411 unique guide RNAs (gRNAs), was used. Lentivirus was produced by transfecting HEK293T cells with the CRISPR library, psPAX2 packaging plasmid, and pMD2.G envelope plasmid using Lipofectamine 3000 (Thermo Fisher Scientific). 143B cells were transduced with the CRISPR library lentivirus. Transduced cells were selected with puromycin (2 µg mL^−1^) for 7 days. Subsequently, mutant cells were treated with cisplatin at gradually increasing concentrations ranging from 0.5 to 2 µm for 10 days, while the control group was treated with an equivalent volume of DMF. Genomic DNA was extracted from both cisplatin‐resistant and untreated control cell. The integrated gRNAs were PCR‐amplified and sequenced using next‐generation sequencing (NGS) on an Illumina platform. MAGeCK (Model‐based Analysis of Genome‐wide CRISPR‐Cas9 Knockout) software was used to identify significantly enriched gRNAs in the cisplatin‐resistant population, pointing to potential cisplatin‐resistant genes.^[^ ^44^ ^]^

### Cell Culture

The 143B (CRL‐8303), HOS (CRL‐1543), and HEK293T (CRL‐3216) cell lines were used in this study. All cell lines were authenticated by STR profiling. All cell lines were obtained from Procell Life Science & Technology Co., Ltd., China. The 143B and 293T cell lines were cultured in Dulbecco's Modified Eagle Medium (DMEM) (Procell Life Science & Technology), while the HOS cell line was cultured in Minimum Essential Medium (MEM) (Procell Life Science & Technology). All media were supplemented with 10% fetal bovine serum (FBS) (NEWZERUM Ltd., New Zealand) and 1% penicillin‐streptomycin (Procell Life Science & Technology). The cells were maintained at 37 °C in 5% CO2 humidified atmospheres. Cells were passaged at 70–80% confluency using TrypLE Express (Procell Life Science & Technology) for detachment.

### Colony Formation Assay

Colonies were formed by seeding cells into 6‐well plates at a density of 1000 cells per well and cultivating them for 7–14 days. The colonies were then fixed with 4% paraformaldehyde for 15 min, stained with 0.5% crystal violet for 30 min, and washed with phosphate‐buffered saline (PBS). The number of colonies was counted using ImageJ software.

### CCK‐8 Assay

Cell proliferation and viability were assessed using the CCK‐8 assay (Beyotime, C0037).^[^ ^45^ ^]^ In the cell proliferation assay, 1000 cells were seeded per well in 96‐well plates, while for the IC50 determination, 5000 cells were seeded per well. At various time points, 10 µL of CCK‐8 reagent was added to each well and incubated at 37 °C for 2 h. Absorbance was measured at 450 nm using a microplate reader (Bio‐Rad). Cell viability was calculated relative to control wells.

### Flow Cytometry

Flow cytometry was used to analyze cell apoptosis. Cells were harvested, washed twice with cold PBS, and resuspended in 1X binding buffer at a concentration of 1×10^6 cells mL^−1^. Annexin V‐FITC and propidium iodide (PI) staining was performed using the Annexin V‐FITC Apoptosis Detection Kit (#40302ES60, YEASEN) according to the manufacturer's instructions. Briefly, 100 µL of the cell suspension was incubated with 5 µL of Annexin V‐FITC and 5 µL of PI for 15 min at room temperature in the dark. After incubation, 400 µL of 1X binding buffer was added to each tube. The samples were then analyzed by flow cytometry using a BD FACSCalibur flow cytometer (BD Biosciences). Data were acquired and analyzed using FlowJo software (FlowJo, USA). Early apoptotic cells were identified as Annexin V‐FITC positive and PI negative, while late apoptotic or necrotic cells were both Annexin V‐FITC and PI positive.

### Transfection of Plasmids

According to the manufacturer's instructions, plasmid transfection was performed using Lipofectamine 2000 (Thermo Fisher Scientific). Briefly, cells were seeded into 6 cm dishes at a density of 5×10^5 cells per dish and cultured overnight to reach 70–80% confluency. The plasmid DNA (5 µg per dish) was diluted in Opti‐MEM medium and mixed with the Lipofectamine 2000 reagent. The DNA‐Lipofectamine complexes were incubated for 20 min at room temperature before being added dropwise to the cells. Cells were collected 72 h after transfection for follow‐up experiments.

### Lentivirus Construction and Transduction

Lentiviral vectors were constructed for gene knockdown. HEK293T cells were seeded in 10 cm dishes and cultured to 70–80% confluency. For each dish, 12 µg of lentiviral plasmid, 6 µg of psPAX2, and 6 µg of pMD2.G were diluted in Opti‐MEM medium and mixed with Lipofectamine 2000. The mixture was incubated for 20 min and then added to the cells. The medium was refreshed 12–16 h post‐transfection. Lentiviral particles were harvested at 48 and 72 h, filtered, and concentrated by ultracentrifugation. The lentiCRISPR v2 (#52 961, Addgene) was provided by Feng Zhang's laboratory. The ERCC6 sgRNA sequences are as follows: sgERCC6#1: F: caccgGAAGGAGTATCGGTCGGTCC, R: cCTTCCTCATAGCCAGCCAGGcaaa; sgERCC6#2: F: caccgTGCCTCCCAGCTCGTTGACG, R: cACGGAGGGTCGAGCAACTGCcaaa.

### Co‐IP

Co‐IP was performed to identify protein‐protein interactions. Cells were lysed in IP lysis buffer containing protease and phosphatase inhibitors. Lysates were incubated with primary antibodies targeting the protein and IgG (#A7007, Beyotime) of interest overnight at 4 °C, followed by incubation with Protein A/G agarose beads (#P2029, Beyotime) for 24 h at 4 °C. Beads were washed extensively with IP lysis buffer, and bound proteins were eluted by boiling in SDS‐PAGE loading buffer. Eluted proteins were separated by SDS‐PAGE and visualized by Coomassie Brilliant Blue staining. Specific bands were excised and subjected to in‐gel digestion with trypsin. The resulting peptides were extracted and analyzed by liquid chromatography coupled to tandem mass spectrometry (LC‐MS/MS).

### GST Pull Down Assay

To determine the interaction domains between ERCC6 and HNRNPM, GST pull‐down assays were performed using a series of truncated constructs. Full‐length and truncated forms of ERCC6 were subcloned to express GST‐tagged proteins, including the full‐length protein and four truncations: D1–2, D1–3, D2–4, and D3–4. (Domain 1 contains amino acids 1–510; Domain 2 contains amino acids 519–750; Domain 3 contains amino acids 843–1002; Domain 4 contains amino acids 1003–1493). Correspondingly, HNRNPM was subcloned to generate GST‐tagged full‐length and two truncated versions: D1–2 and D2–3(Domain 1 contains amino acids 1–150; Domain 2 contains amino acids 151–608; Domain 3 contains amino acids 609–729). All constructs were verified by sequencing. Recombinant plasmids were transformed into E. coli BL21 (DE3) cells (#TsingkeBiotechnologyCo, TSC‐E01), and protein expression was induced with 0.5 mm IPTG (#MCE,367‐93‐1) at 16 °C for 12 h. The bacterial lysate obtained from sonication is applied to GST purification magnetic beads (#P2251, BeyoGold) and incubated with mixing at 4 °C for 4–6 h, then recombinant HIS‐tagged proteins were added, followed by further incubation at 4 °C with mixing for 2 h. After extensive washing to remove nonspecific interactions, bound complexes were eluted in SDS‐PAGE loading buffer, resolved by SDS‐PAGE, and subjected to WB. An anti‐HIS antibody (#Abbkine, A02050) was used to detect interacting HIS‐tagged proteins, and anti‐GST antibody (#ZenBio,300 195) was used as a loading control for the immobilized GST fusion proteins.

### Molecular Docking

Protein–protein docking between ERCC6 and HNRNPM was conducted to investigate their potential interaction interface. The crystal structure of ERCC6 (PDB ID: 4CVO) and HNRNPM (PDB ID: 2DGV) were obtained from the Protein Data Bank. Prior to docking, PyMOL (version 2.6) was used to remove water molecules and non‐essential ligands, and the cleaned structures were saved in PDB format. Docking was performed using the ZDOCK web server, which applies a rigid‐body FFT‐based algorithm to generate docking poses. The top‐ranked docking model was selected based on ZDOCK scores and submitted to the PDBePISA server to analyze predicted interaction residues and estimate binding free energy.

### Mouse Xenograft Assay

Mouse xenograft assays were conducted to evaluate the effect of gene knockdown on cisplatin sensitivity in vivo. Two independent experiments were performed, each including four groups: control, knockdown, control with cisplatin, and knockdown with cisplatin. For each experiment, 5‐week‐old female BALB/c nude mice were subcutaneously injected with 5×10^6 143B cells transduced with either control or knockdown lentiviral vectors. Animals were assigned to experimental groups using simple randomization. Responses were then scored by an experimenter blinded to injection condition and experimental cohort. Once tumors reached an average volume of 50–100 mm^3, mice in the treatment groups received intraperitoneal injections of cisplatin at a dose of 3 mg kg^−1^ twice a week. The control groups received saline injections. Tumor size was measured twice weekly using calipers, and tumor volume was calculated using the formula: (length × width^2)/2. Mice were monitored for weight loss and general health. At the end of the treatment period, mice were sacrificed, and tumors were excised, weighed, and subjected to further analysis, including IHC for Ki‐67 and cleaved caspase‐3 (Approval No. LYF20240227).

### Full‐Length Transcriptome Sequencing and Variable Splicing Analysis

Full‐length transcriptome sequencing was performed using Oxford Nanopore Technologies (ONT) to analyze nine samples, including three control samples, three ERCC6 knockdown samples, and three HNRNPM knockdown samples. Nanopore sequencing is a next‐generation single‐molecule real‐time sequencing technology based on nanopore electrical signal detection. The experimental workflow followed the standard protocol provided by ONT, including sample quality assessment, library preparation, library quality assessment, and sequencing.^[^ ^46^ ^]^ Raw sequencing reads were base‐called and processed using the ONT software suite. Full‐length transcriptomes were reconstructed, and differential splicing events were identified. The results were visualized using Integrative Genomics Viewer (IGV), allowing for detailed inspection of alternative splicing events and confirmation of identified splice variants.

### Agarose Gel Electrophoresis

Agarose gel electrophoresis was used to analyze PCR products and confirm alternative splicing events. A 1.5% agarose gel was prepared using TBE buffer (Tris‐borate‐EDTA).^[^ ^47^ ^]^ Agarose powder was dissolved in TBE buffer by heating, and ethidium bromide (0.5 µg mL^−1^) was added to the molten agarose for DNA staining. PCR products were mixed with 6X loading dye. The samples were loaded into the wells of the agarose gel, and electrophoresis was carried out in TBE buffer at 100 V for 45–60 min. DNA bands were visualized under UV light using a gel documentation system (Bio‐Rad).^[^ ^48^ ^]^

### ERCC6 and HNRNPM Correlation Analysis

To assess the correlation between ERCC6 and HNRNPM expression, RNA sequencing data from publicly available datasets, including TARGET (Osteosarcoma), TCGA (Sarcoma), GSE21257 (Osteosarcoma), and GSE218035 (Osteosarcoma), were analyzed. Expression levels of ERCC6 and HNRNPM were extracted from these datasets. Pearson correlation (Correlation tests: Pearson's) coefficients were computed to evaluate the strength and direction of the relationship between ERCC6 and HNRNPM expression across the various cohorts. Correlation significance was determined by *P* values, and scatter plots were generated to visually represent the associations between ERCC6 and HNRNPM expression levels within each dataset.

### Statistical Analysis

Statistical analyses were performed using GraphPad Prism version 9.0. Data were presented as mean ± standard deviation (SD). Differences between groups were assessed using one‐way or two‐way ANOVA followed by Tukey's post hoc test for multiple comparisons. For comparisons between the two groups, the student's t‐test was used. The correlation between gene expression and clinical parameters was analyzed using Pearson's correlation coefficient. Statistical significance was set at *p* < 0.05. For the RNA sequencing data, differential gene expression analysis was conducted using DESeq2, with a *p*‐value < 0.05 considered significant. Functional enrichment analysis of differentially expressed genes and alternative splicing events was performed using Gene Ontology (GO) and Kyoto Encyclopedia of Genes and Genomes (KEGG) pathways. All experiments were repeated at least three times independently to ensure reproducibility. This comprehensive statistical approach ensured the robustness and reliability of the experimental findings.

## Conflict of Interest

The authors declare no conflict of interest.

## Author Contributions

R.X. performed conceptualization, data curation, formal analysis, validation, funding acquisition, investigation, visualization, methodology, wrote the original draft and editing. S.Z. performed conceptualization, data curation, formal analysis, validation, investigation, visualization, and methodology. W.Z. performed Investigation, methodology, and provided resources. C.T. performed investigation, methodology, and provided resources. H.W., L.W., and T.L. performed investigation and methodology. X.R. performed conceptualization, data curation, supervision, funding acquisition, project administration, wrote, reviewed, and edited the draft and provided resources. Z.L. performed conceptualization, data curation, formal analysis, supervision, funding acquisition, investigation, visualization, project administration, wrote, reviewed, and edited the draft, and provided resources.

## Supporting information



Supporting Information

Supplemental Table 1

Supplemental Table 2

Supplemental Table 3

Supplemental Table 4

## Data Availability

The authors acquired the gene expression data of OS patients from Therapeutically Applicable Research To Generate Effective Treatments (TARGET, https://ocg.cancer.gov/programs/target) and sarcoma patients from The Cancer Genome Atlas (TCGA, https://portal.gdc.cancer.gov/). Datasets GSE21257^[^ ^49^ ^]^ and GSE218035^[^ ^10^ ^]^ could be obtained from Gene Expression Omnibus (GEO, http://www.ncbi.nlm.nih.gov/geo/). Additionally, the datasets used and analyzed during the current study are available from the corresponding authors upon reasonable request.
