# Supplementary material for: A Dual Approach with Organoid and CRISPR Screening Reveals ERCC6 as a Determinant of Cisplatin Resistance in Osteosarcoma
Source: Adv Sci (Weinh). 2025 Jun 6;12(28):2500632. doi: 10.1002/advs.202500632 (PMC12302615; doi:10.1002/advs.202500632)

Supporting Information

**Title**

A Dual Approach with Organoid and CRISPR Screening Reveals ERCC6 as a Determinant of Cisplatin Resistance in Osteosarcoma

*Ruiling Xu^1,2^, Sai Zhu^1,2^, Wenchao Zhang^1,2^, Haodong Xu^1,2^, Chao Tu^1,2^, Honghui Wang^3^, Lu Wang^1,2^, Na He^1,2^, Tang Liu^1^, Xiaoning Guo^1^, Xiaolei Ren^1,2^*, Zhihong Li^1,2^**

**List of Supplementary Materials**

Fig S1 to S5 for multiple supplementary figures

Tables S1 to S4 for multiple supplementary tables


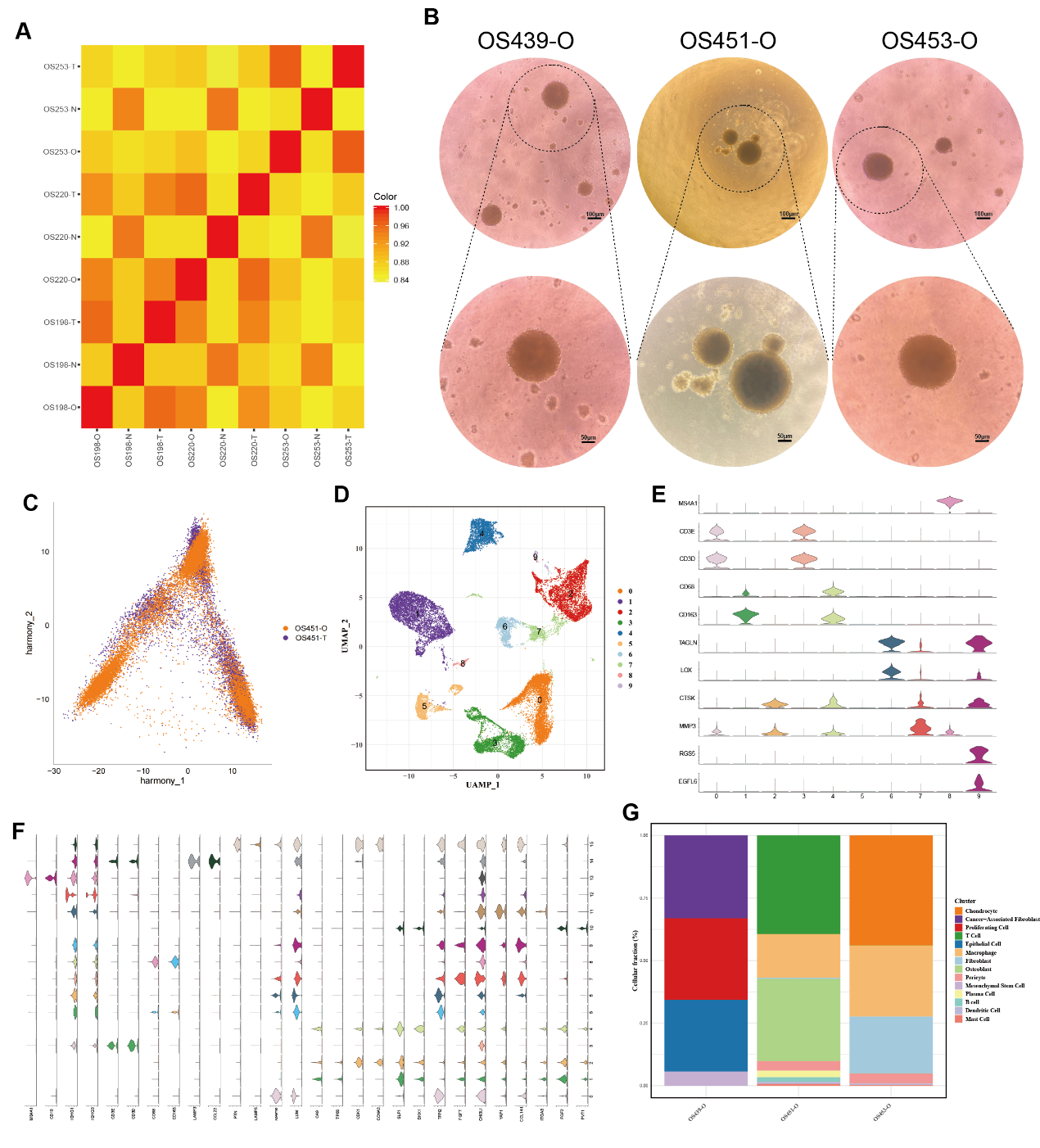


**Figure S1.**

**Correlation Heatmap and Single-cell Analysis of OSOs**

**(A).** Correlation heatmap showing hierarchical clustering of normal tissue, tumor tissue, and OSOs from three patients, demonstrating the relationships between the samples.

**(B).** Light microscopy images of three OSOs, highlighting their morphology.

**(C).** Harmony plot of single-cell RNA-seq data from primary tumors and their corresponding OSOs after batch correction.

**(D).** UMAP plot after batch correction, showing the overlap in cell types between primary tumors and OSOs.

**(E).** Annotated UMAP plots based on cell type markers, revealing the presence of all major tumor cell types except vascular-related cells.

**(F-G).** Combined UMAP plots demonstrating the diverse cellular composition in OSOs from three patients, with distinct clusters representing different cell types.


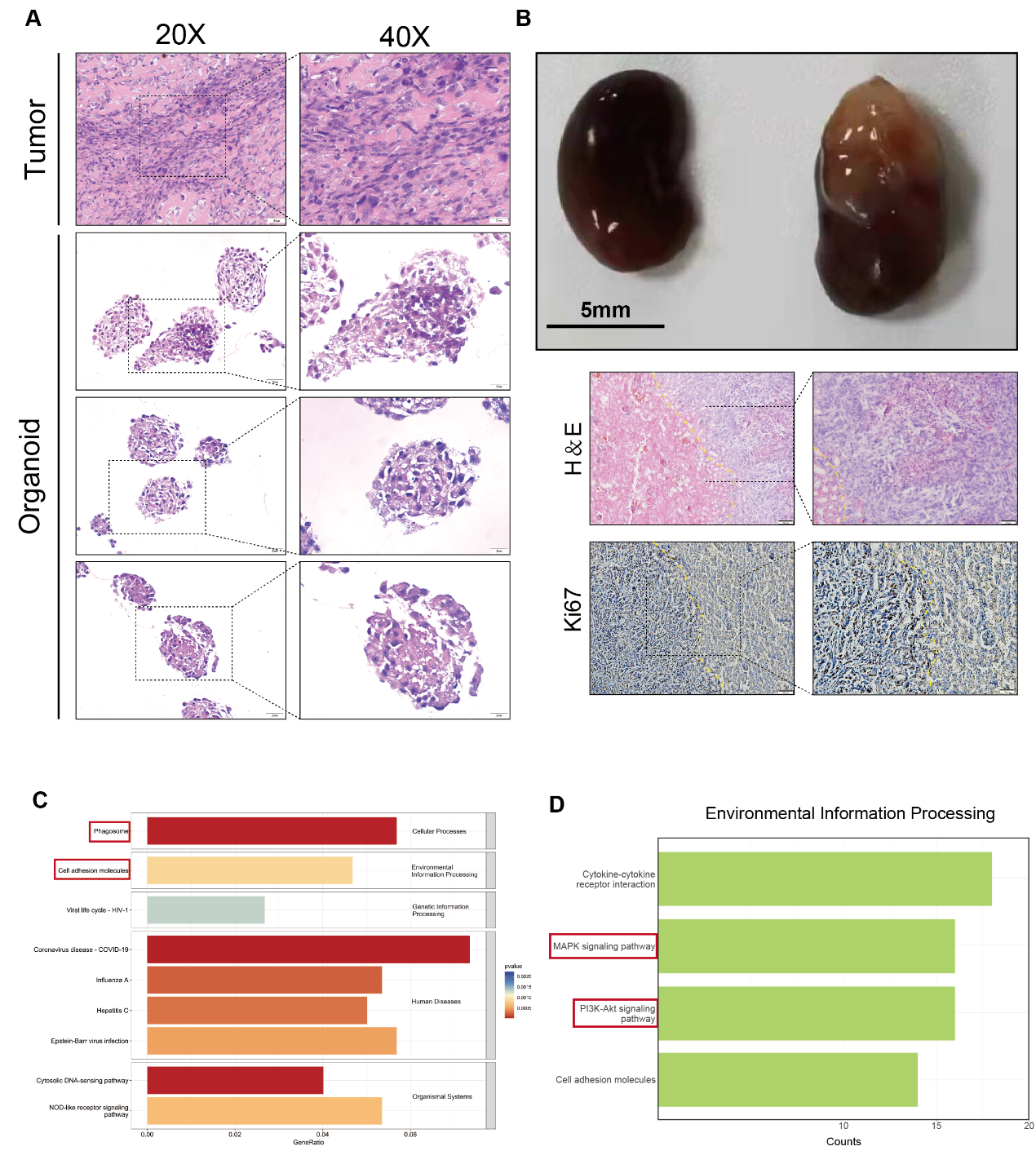


**Figure. S2.**

**Characterization and functional validation of OSOs and cisplatin resistance pathways.**

**(A).** H＆E staining comparison between tumor sections and corresponding organoid sections, showing histopathological similarities.

**(B).** Mouse renal capsule tumor formation assay of OSOs, with human Ki67 IHC staining to highlight proliferation. A clear boundary between tumor tissue and normal tissue is visible.

**(C-D).** KEGG pathway analysis of differentially expressed genes in cisplatin-resistant OSOs, highlighting enrichment in the MAPK and PI3K/AKT pathways.


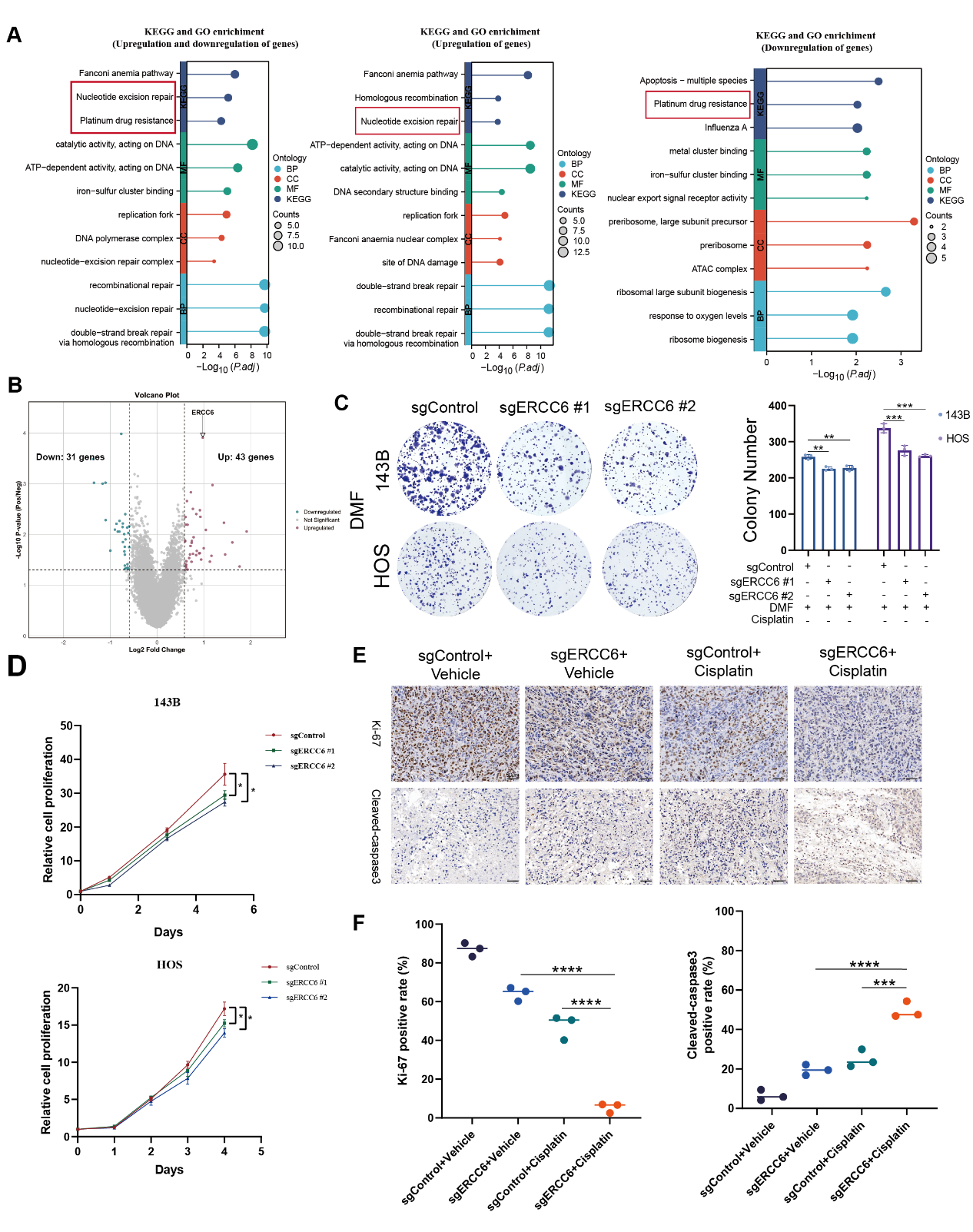


**Figure. S3.**

**Identification of ERCC6 and In Vivo and In Vitro Analysis**

**(A).** KEGG and GO enrichment analyses of significant genes identified from the CRISPR screen.

**(B).** Volcano plot of the CRISPR screen process used to identify cisplatin resistance genes, leading to the discovery of ERCC6.

**(C).** Clonogenic assay results without cisplatin treatment, showing colony formation in control and knockdown cells.

**(D).** Growth curves showing the effect of ERCC6 knockout on cell proliferation under cisplatin-free conditions

**(E-F).** IHC analysis of Ki67 and cleaved caspase-3 staining in mouse tumor sections, revealing proliferation and apoptosis levels in tumors with and without cisplatin treatment.


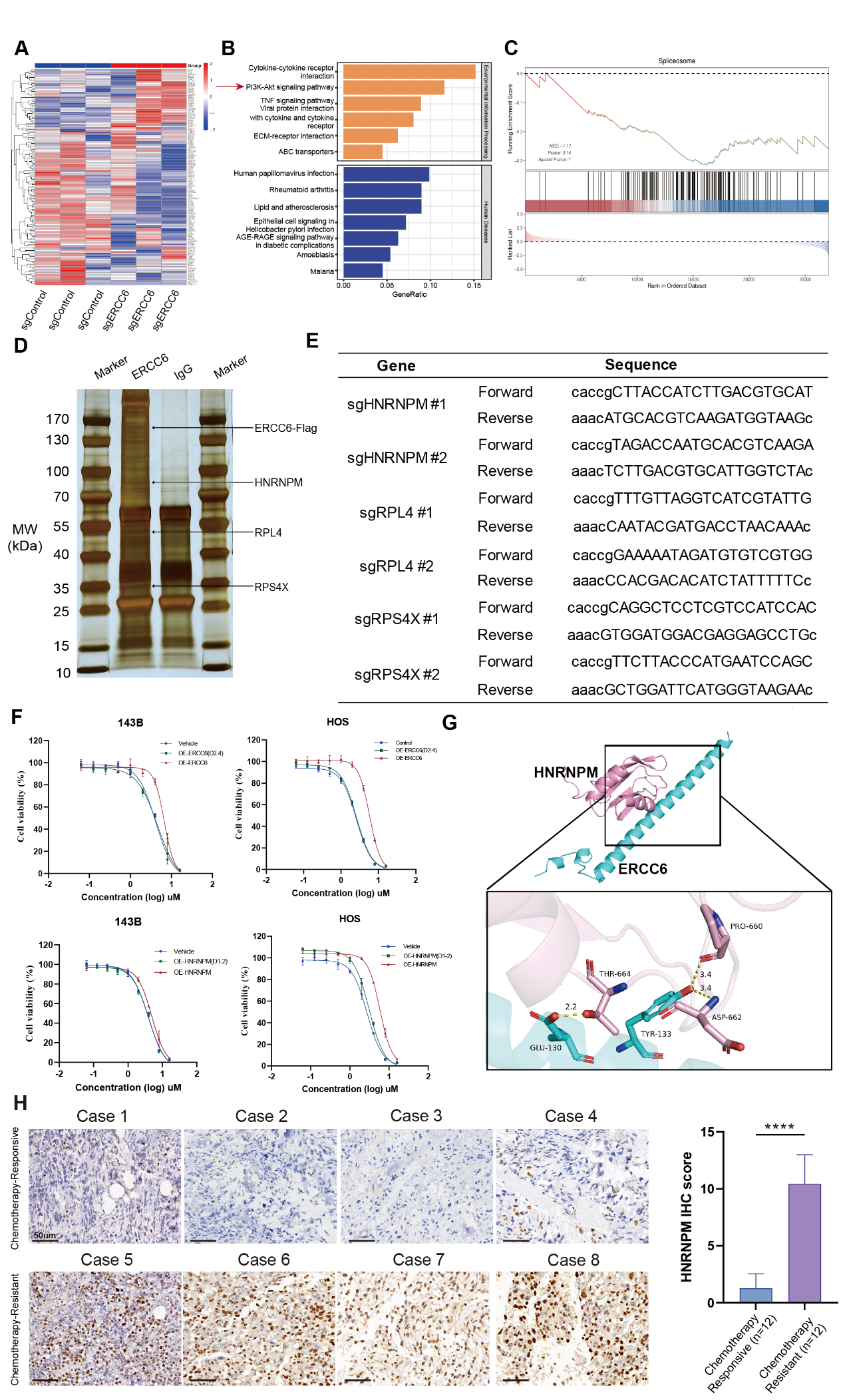


**Figure. S4.**

**Gene Expression and Protein-Protein Interaction of ERCC6**

**(A).** Heatmap of differential gene expression analysis in ERCC6 knockout cells, showing downregulation of key pathways and genes.

**(B)** KEGG pathway analysis shows significant enrichment of the PI3K/AKT pathway in ERCC6 knockout cells

**(C).** KEGG pathway analysis of ERCC6 knockdown cells, highlighting the impact on the splicing pathway.

**(D).** Silver staining results from Co-IP experiments identifying ERCC6-binding proteins.

**(E).** Sequences of sgRNAs targeting potential ERCC6-binding proteins used for CRISPR screens.

**(F)** IC50 assays of full-length and truncated ERCC6/HNRNPM constructs.

**(G)** Molecular docking model showing the predicted interaction interface between ERCC6 and HNRNPM, with key binding residues highlighted.

**(H)** IHC staining of HNRNPM in chemotherapy-resistant and -responsive osteosarcoma tissues.


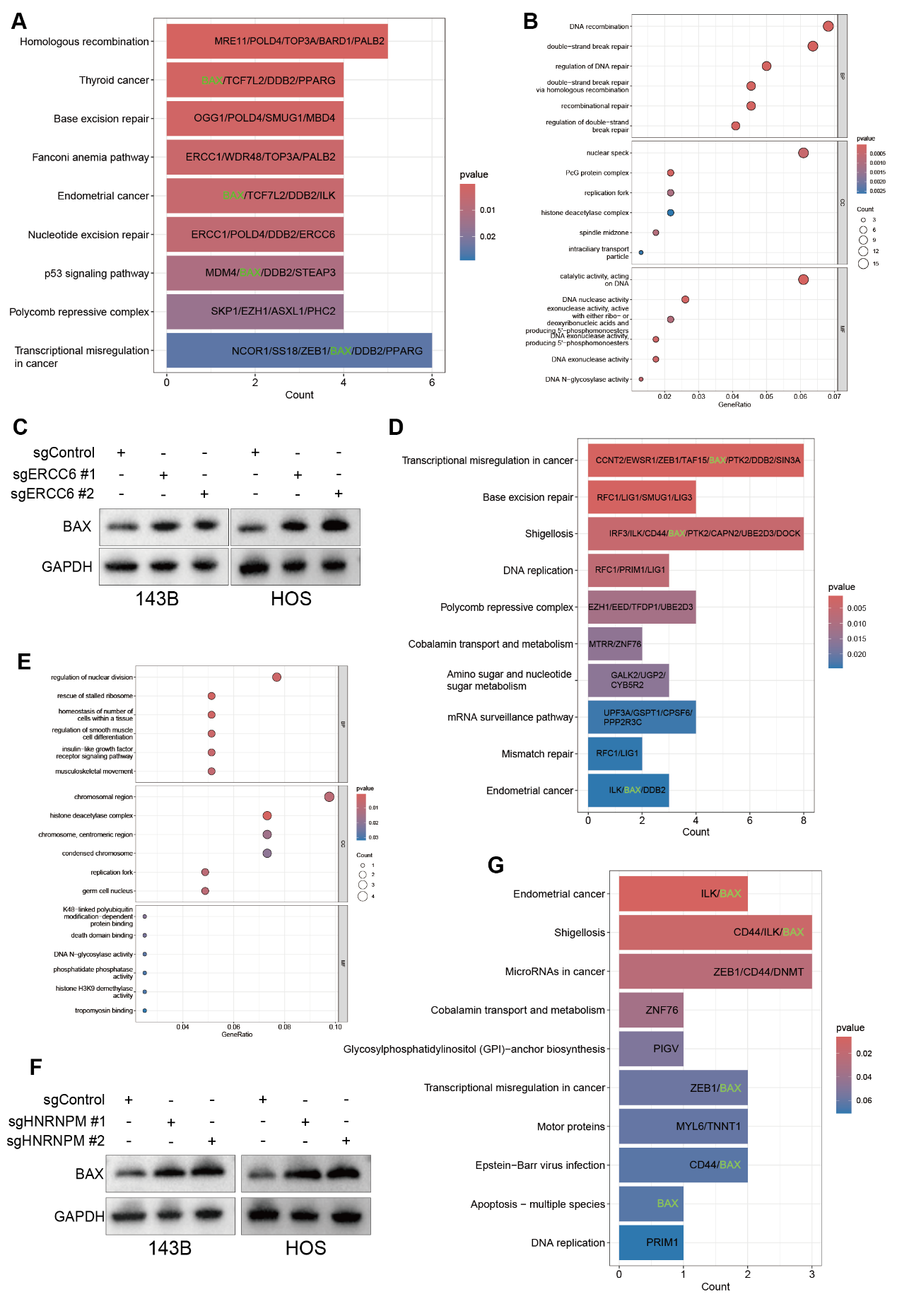


**Figure. S5.**

**Differential Splicing and BAX Expression in Knockdown Cells**

**(A-B).** KEGG and GO analysis of differentially spliced genes in ERCC6 knockdown cells, highlighting BAX’s role in multiple pathways.

**(C).** WB analysis showing increased BAX expression in ERCC6 knockdown cells.

**(D-E).** KEGG and GO analysis of splicing changes in HNRNPM knockdown cells, highlighting BAX as a critical regulator.

**(F).** WB confirming increased BAX expression in HNRNPM knockdown cells.

**(G).** KEGG enrichment of shared splicing events between ERCC6 and HNRNPM knockdowns, identifying BAX as a central gene.

**Supplementary Tables**

Tables S1 to S4 for multiple supplementary tables

**Table S1** Composition of organoid culture medium

**Table S2** TOP 10 marker genes for annotation of sing-cell sequencing

**Table S3** The primer sequences used for RT-qPCR

**Table S4** The raw data of CO-IP

**Figure 2I**

143B


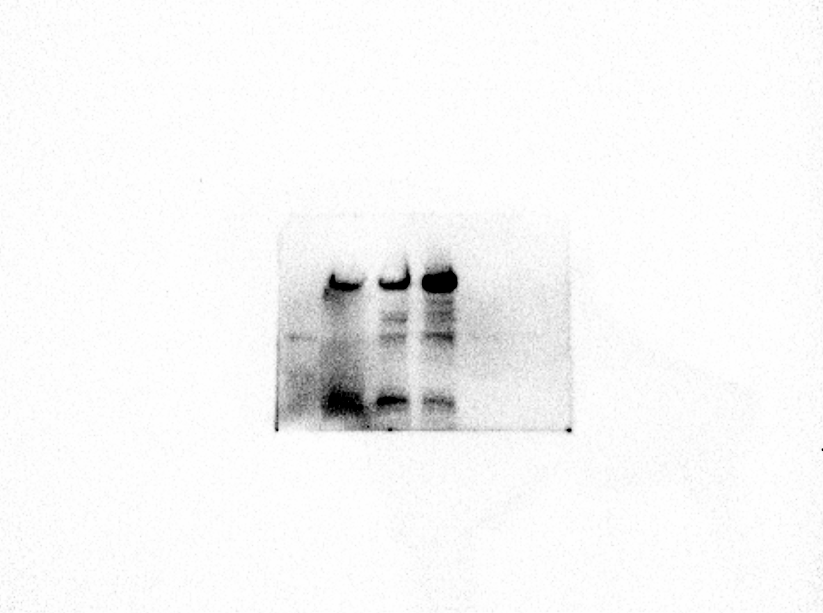

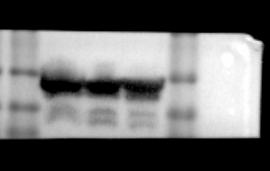


HOS


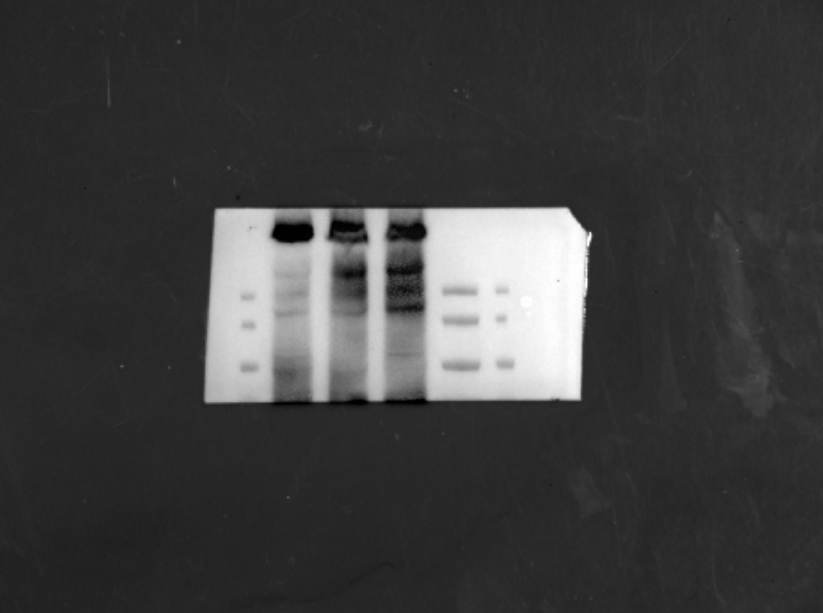

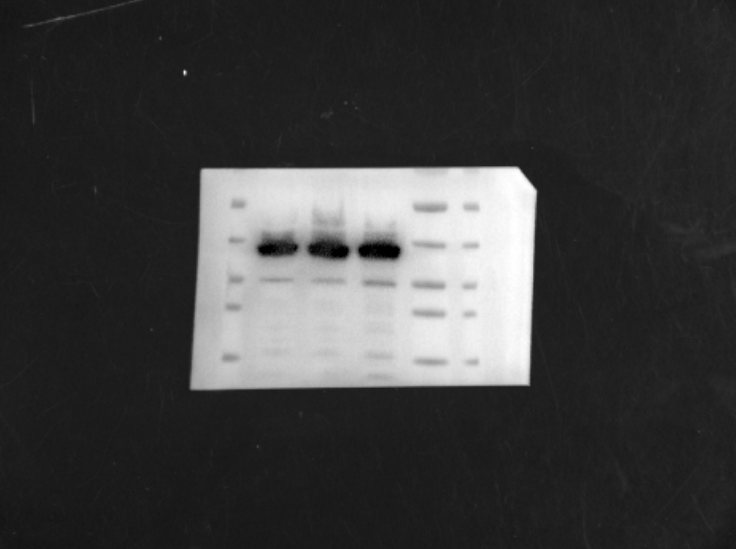


**Figure 3A**

143B

**
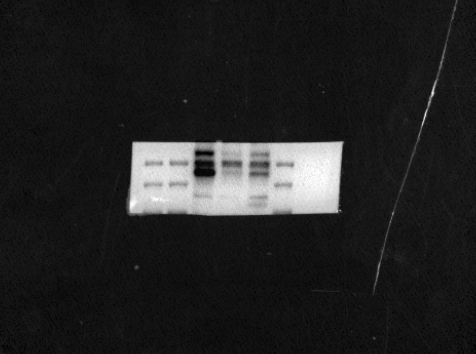

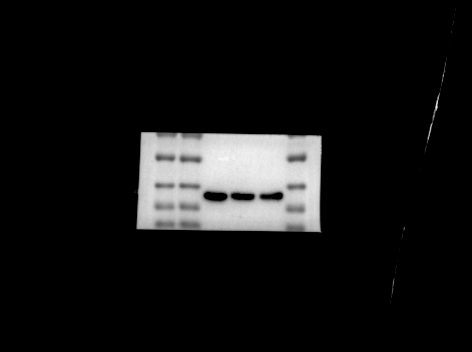
**

HOS

**
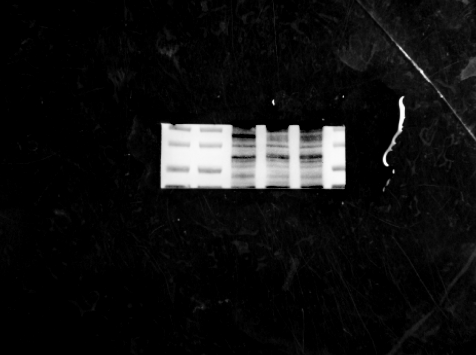

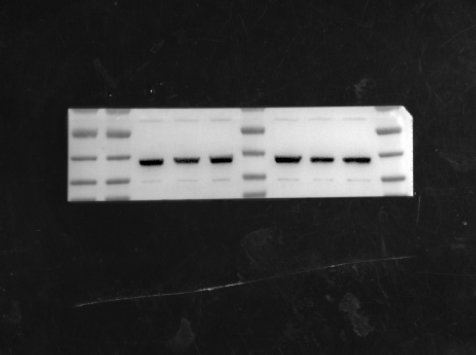
**

**Figure 3F**


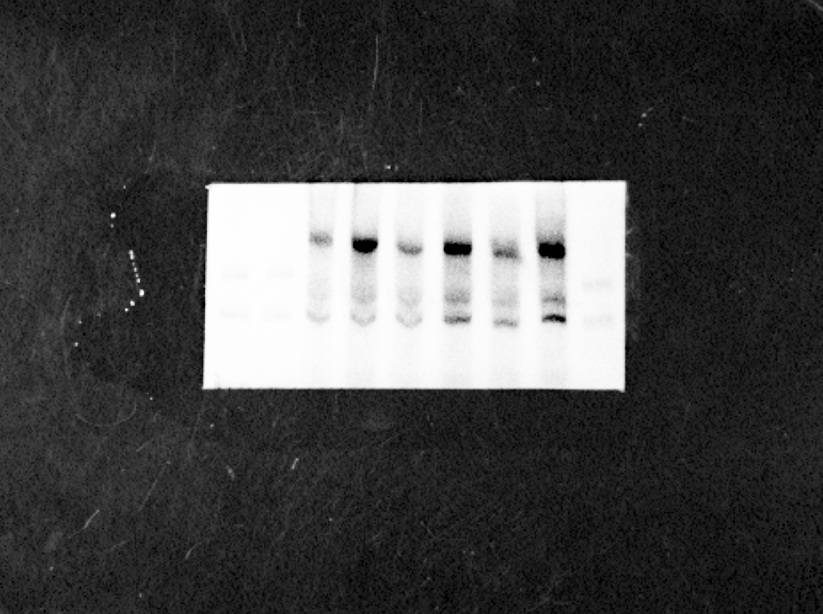

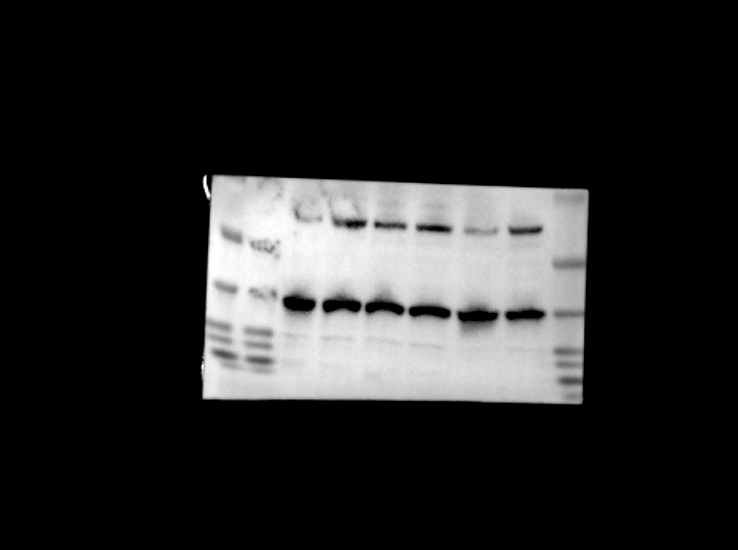


**Figure 3H**

143B


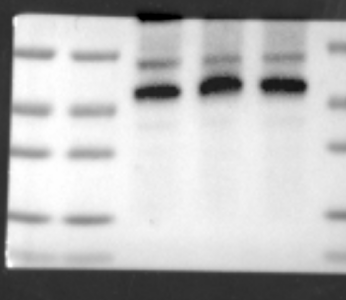

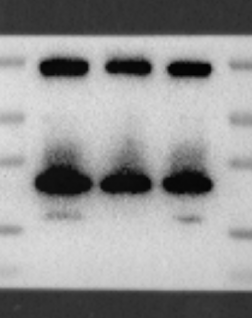


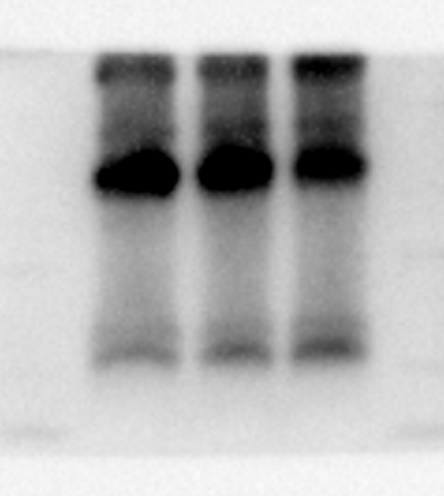

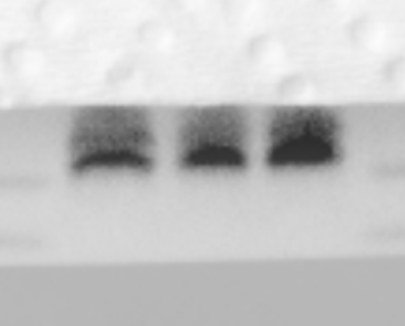

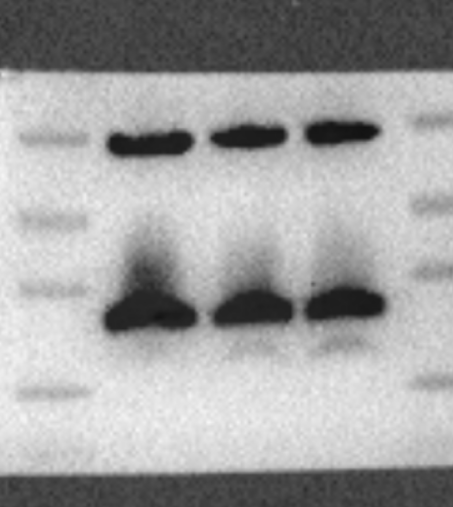


HOS


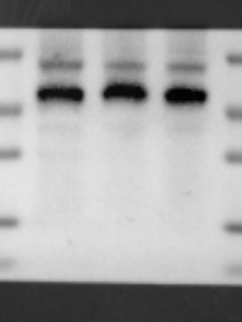

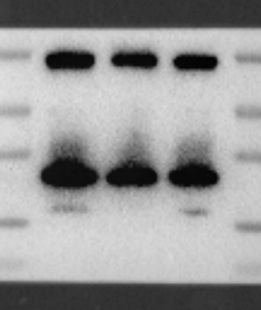


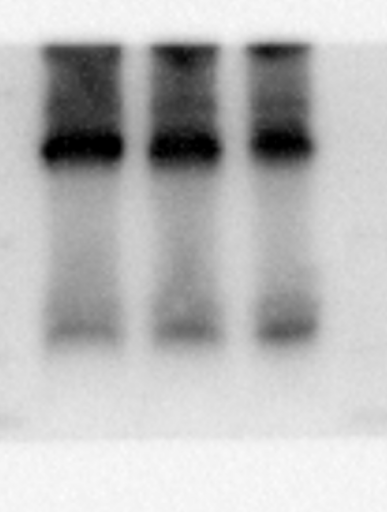

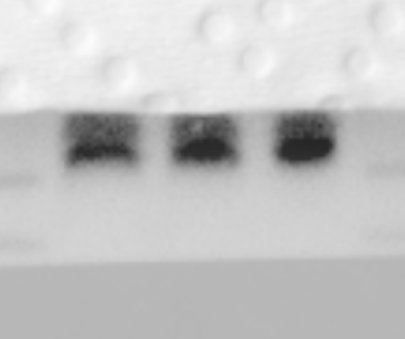

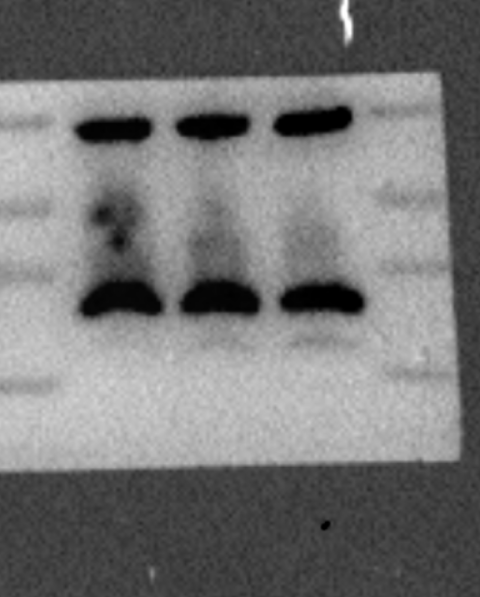


Figure 4E

143B

ERCC6


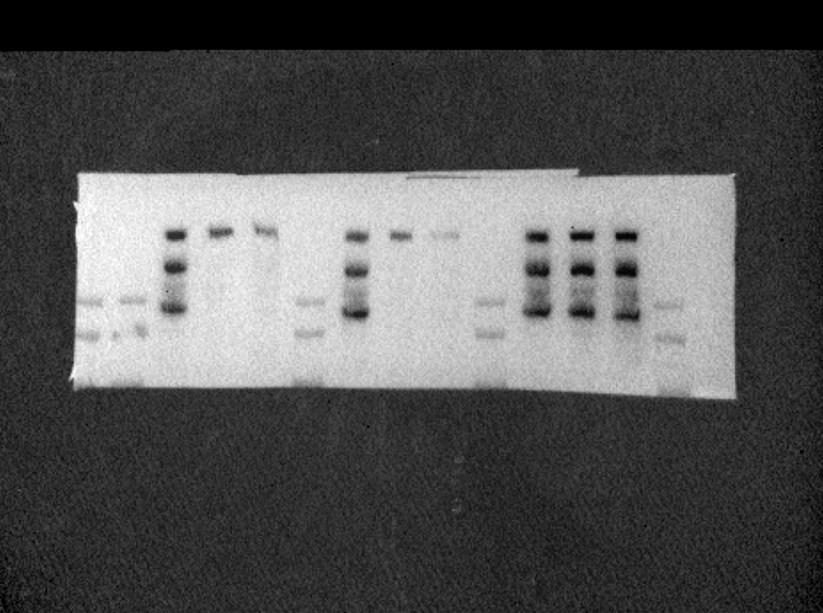


AKT


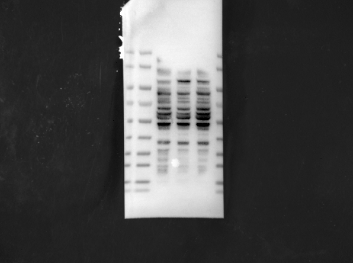


p-AKT


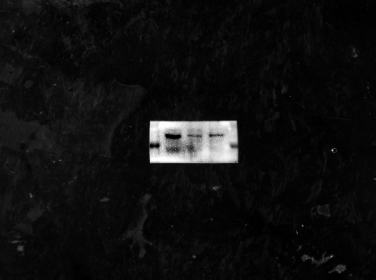


GAP


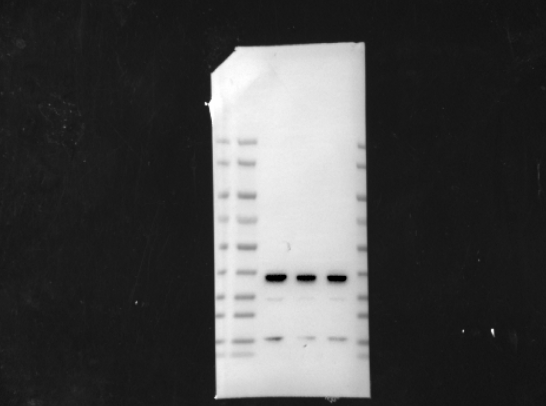


HOS

ERCC6


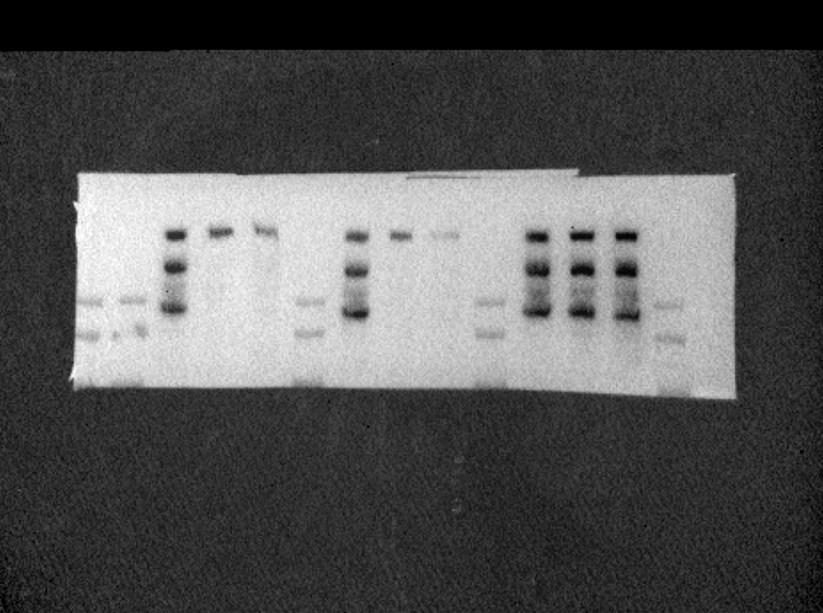


p-AKT


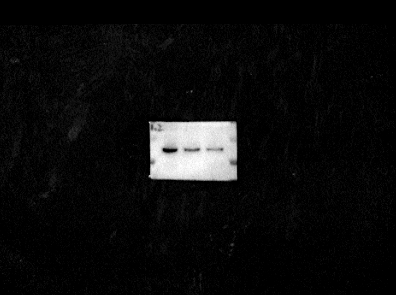


AKT


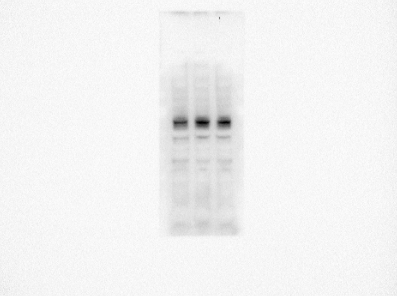


GAPDH


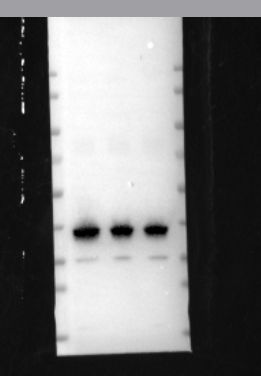


Figure 4E


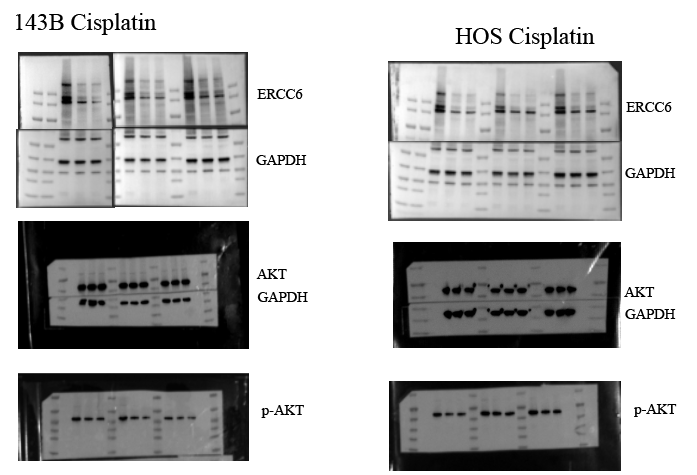


Figure 41

293T


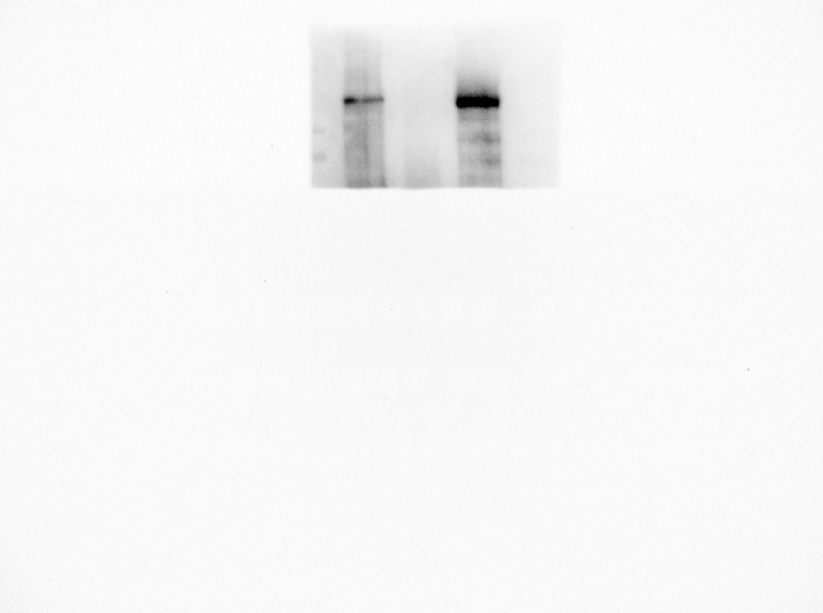

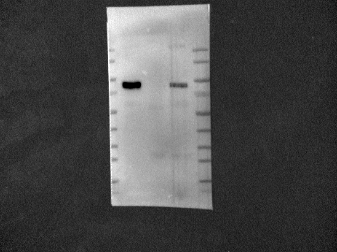


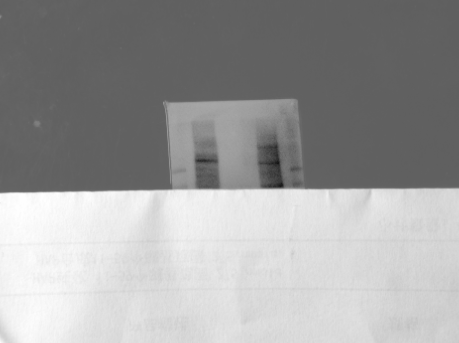

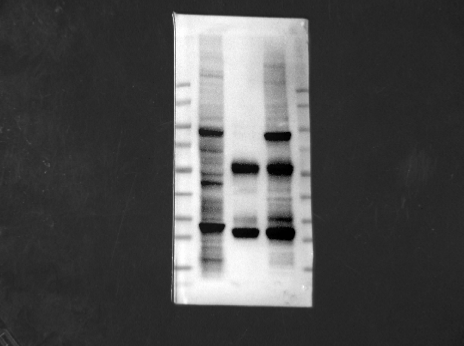


143B


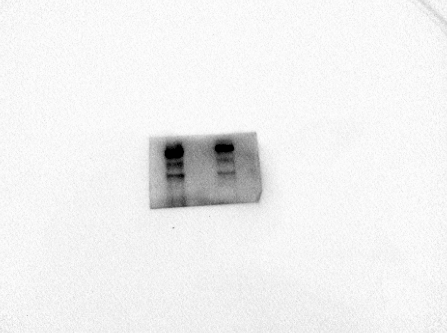

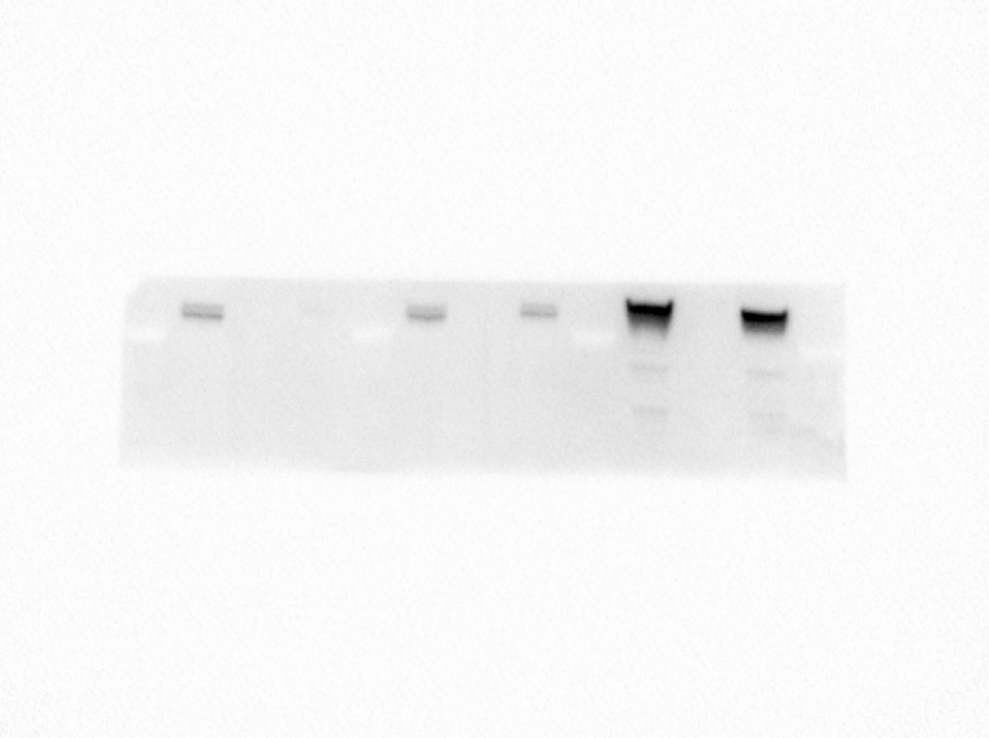


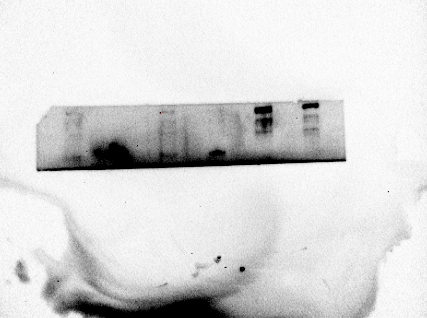

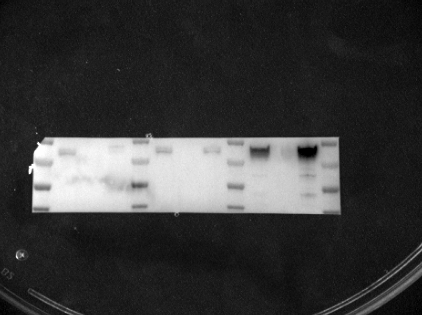


Figure 4J


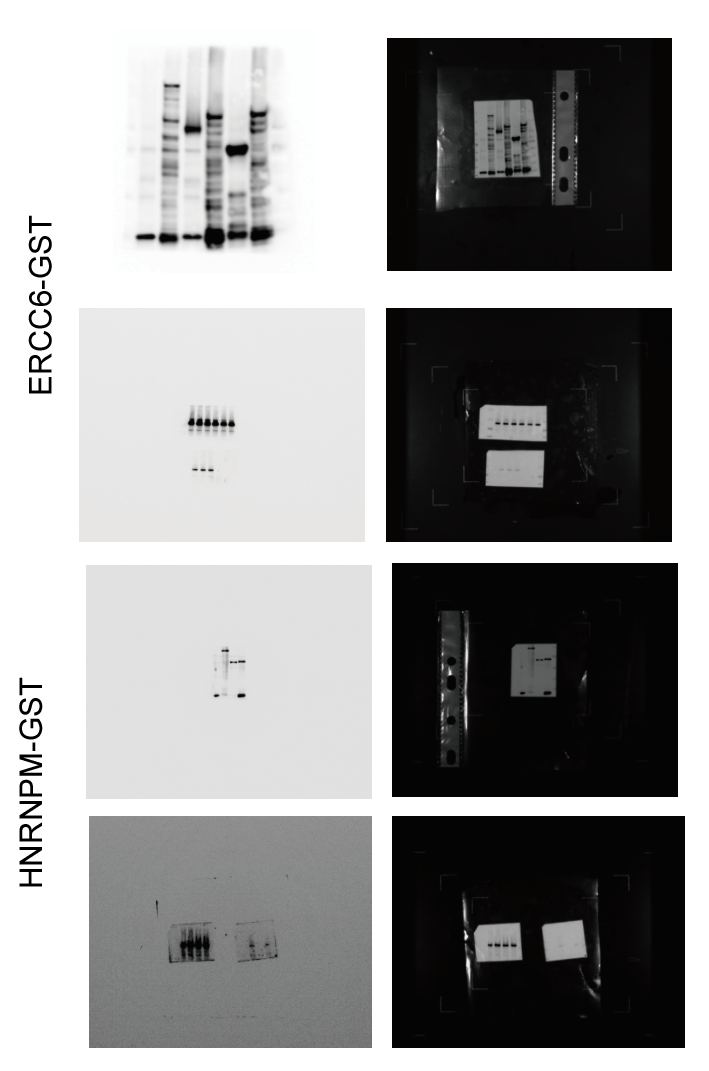


Figure 5A

143B


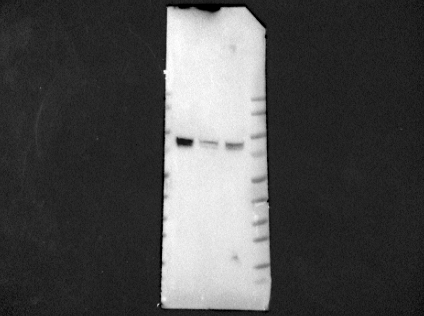

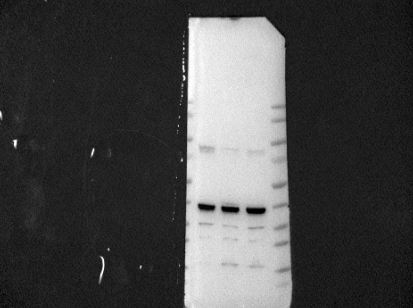


HOS


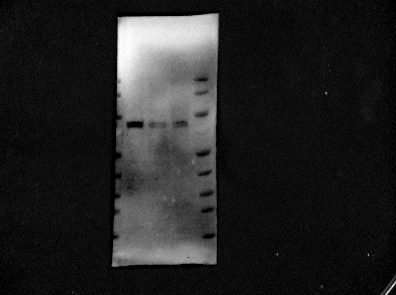

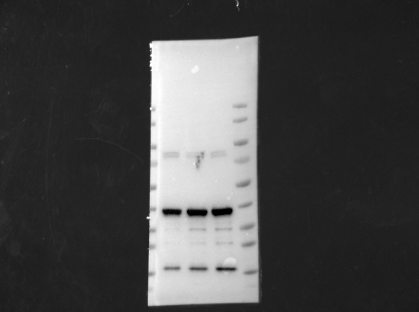


Figure 6A

143B-1


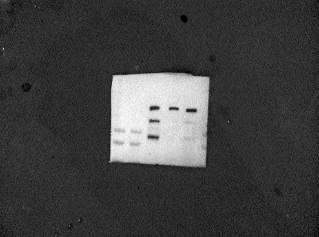

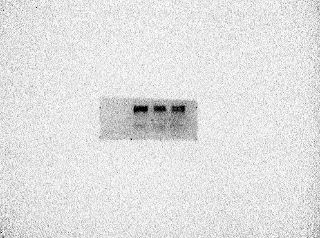

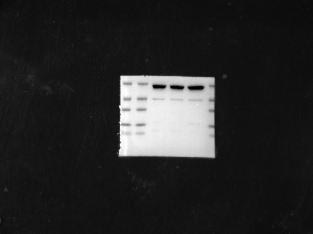


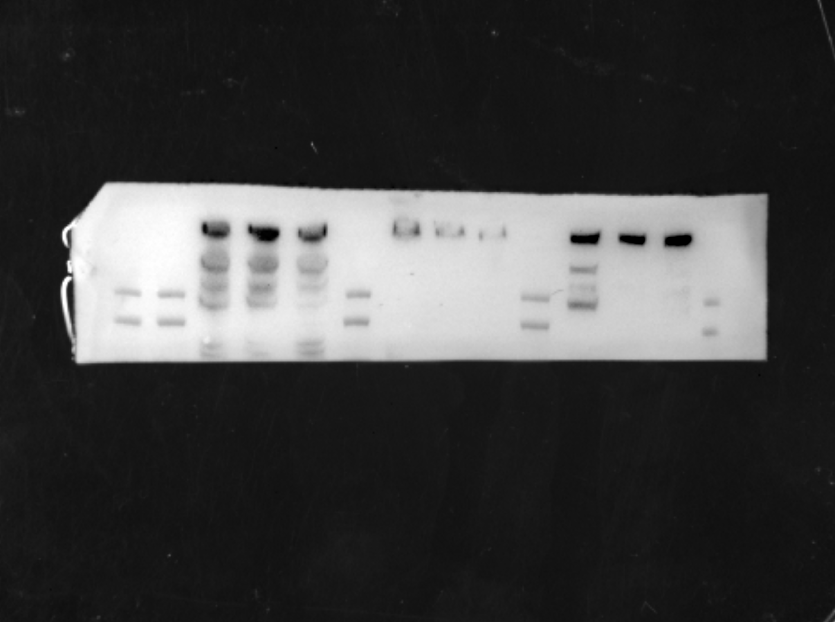

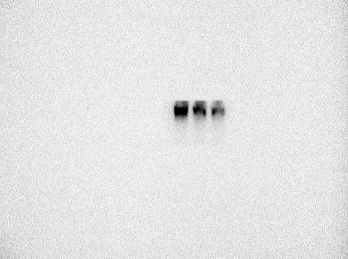

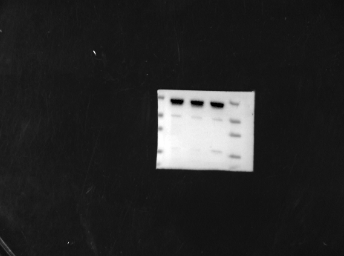


143B-2


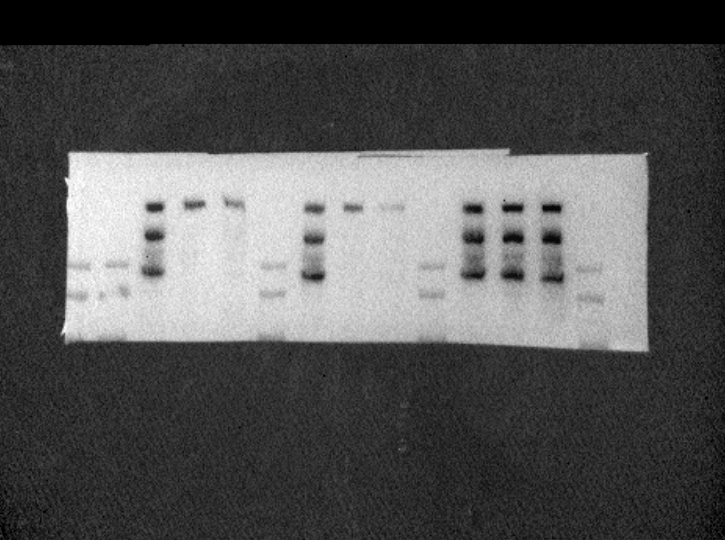

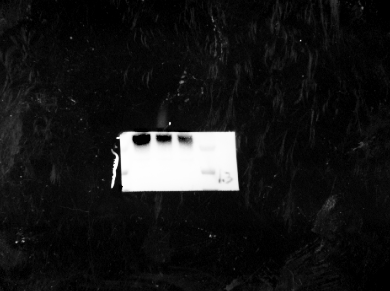

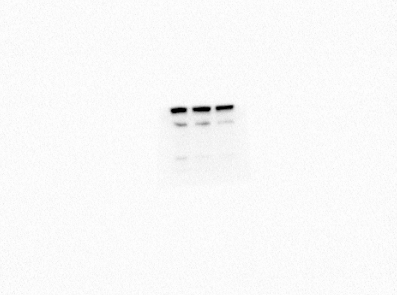


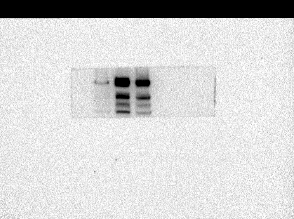

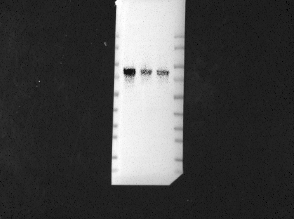

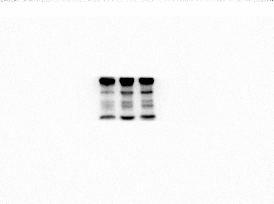


HOS-1


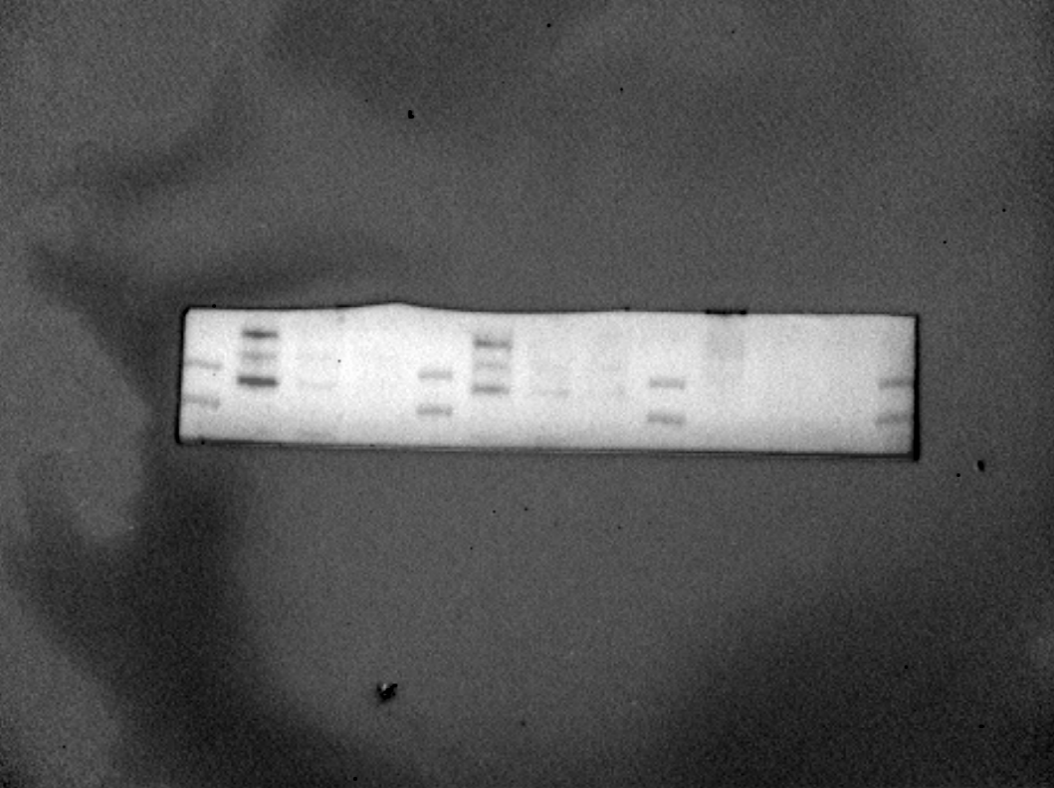

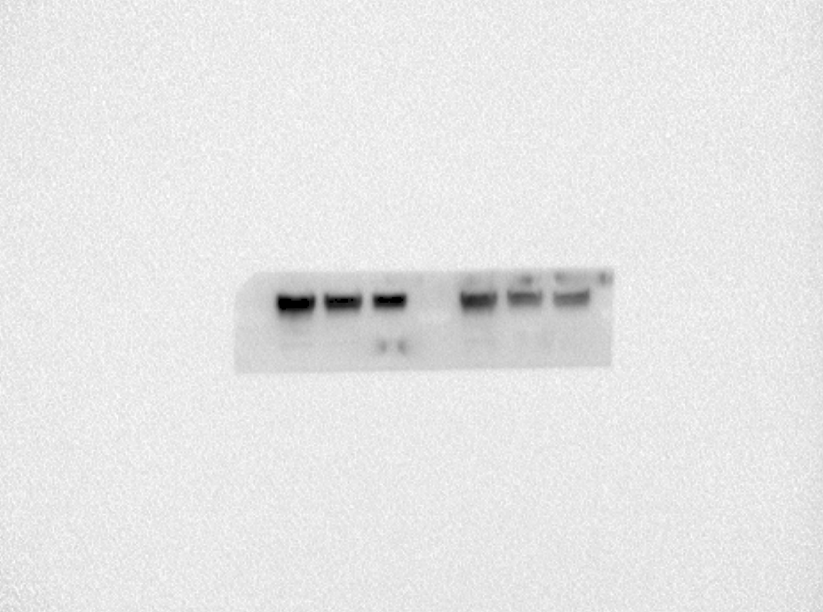

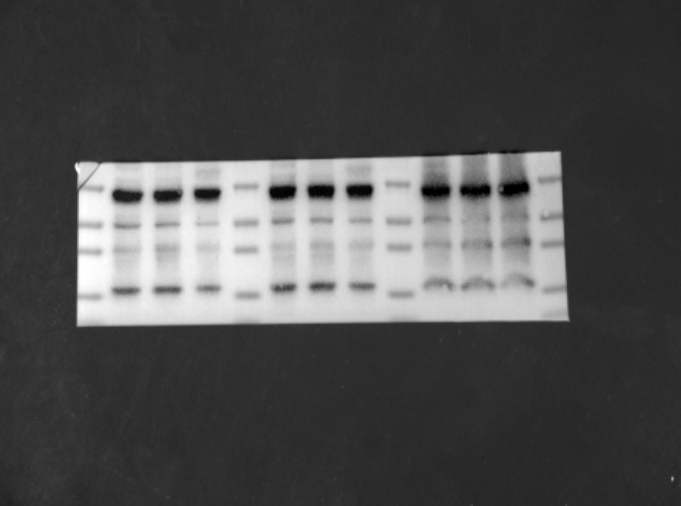


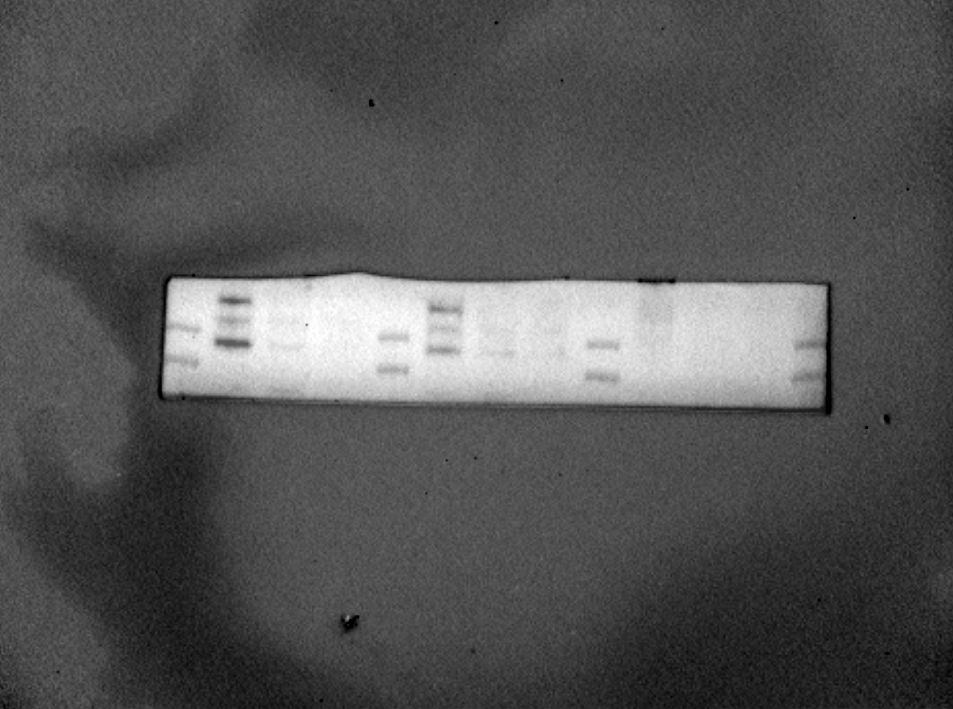

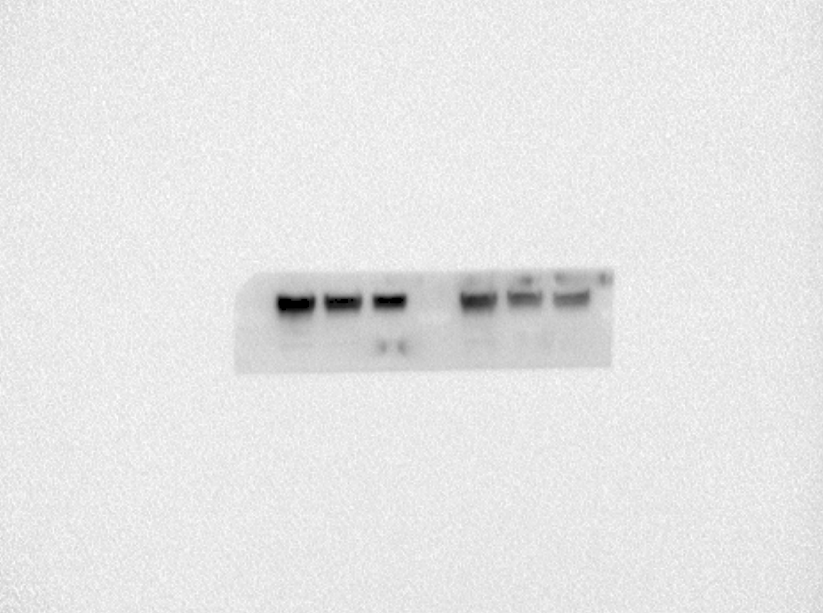

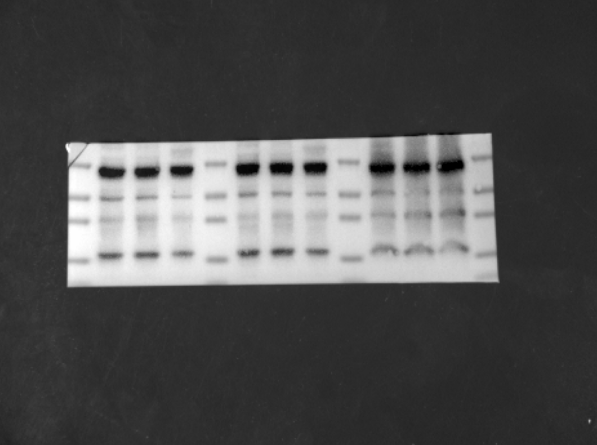


HOS-2


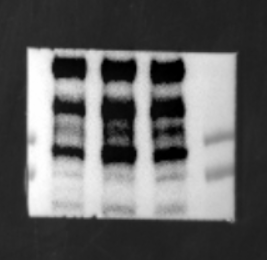

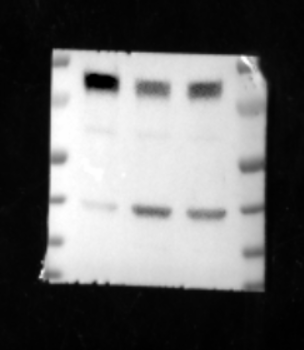

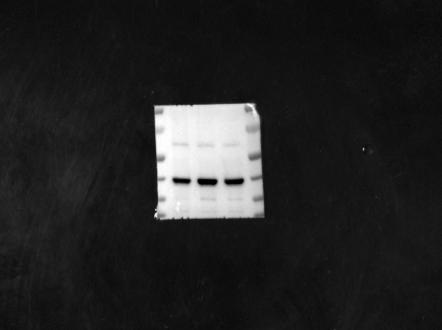


Figure 6F

143B


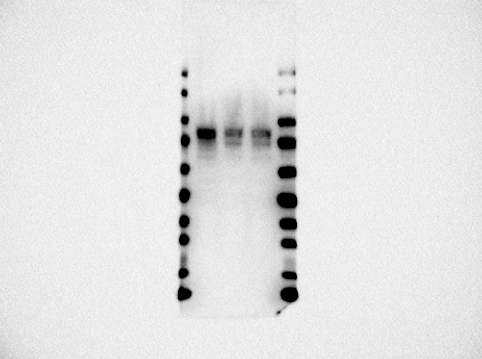

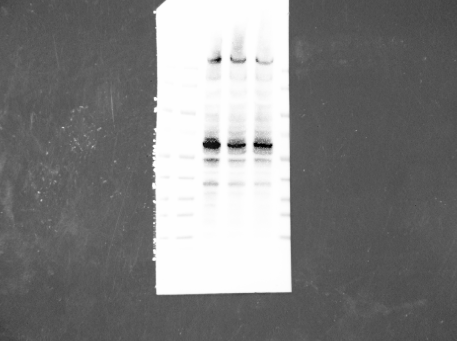


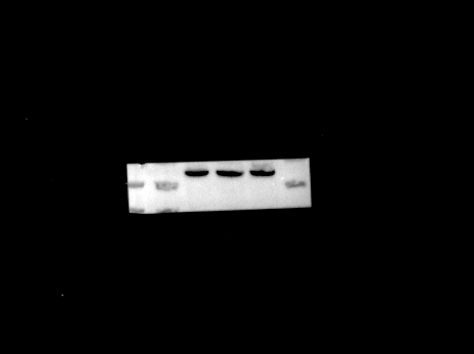

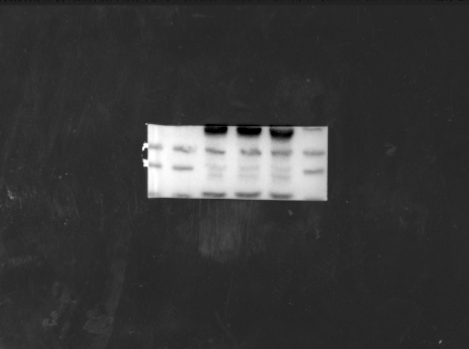


HOS


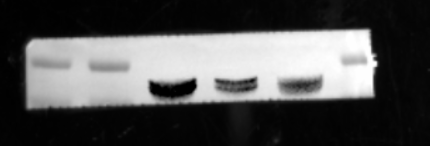

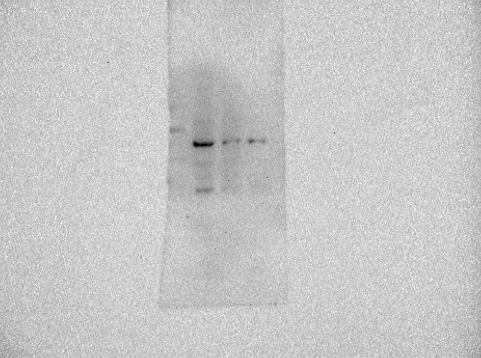


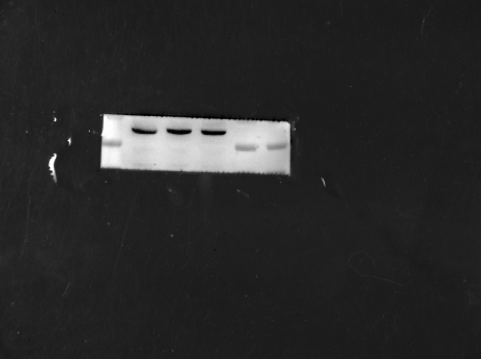

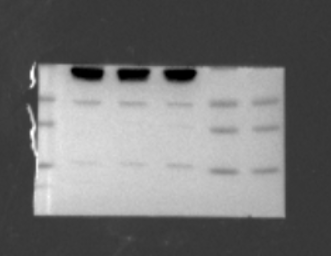


Figure 6F


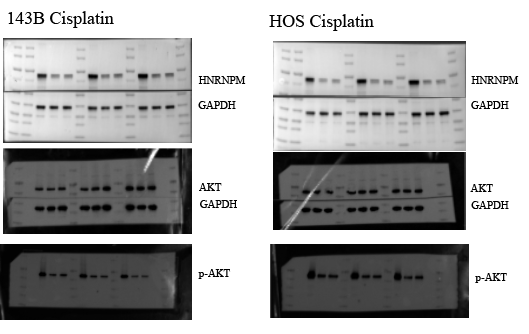


Figure 6G

143B


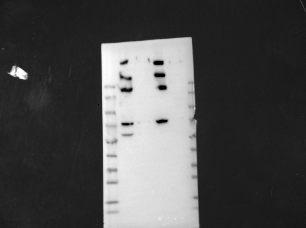

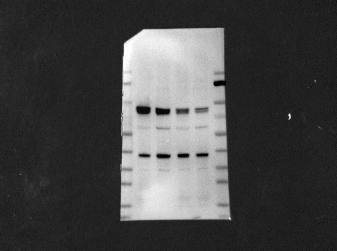

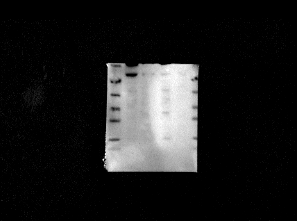

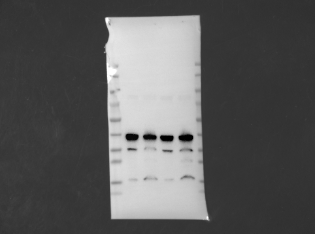


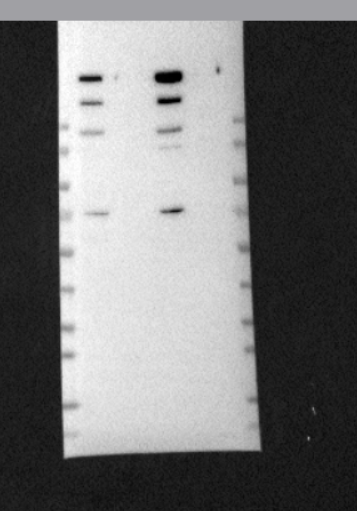

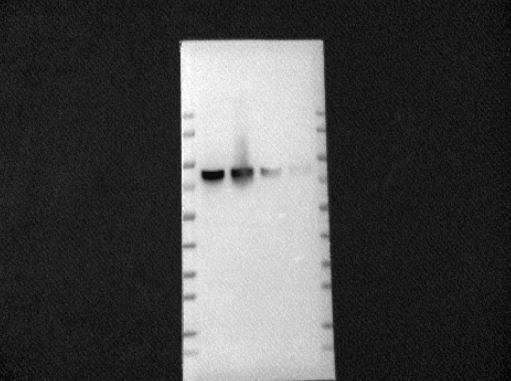

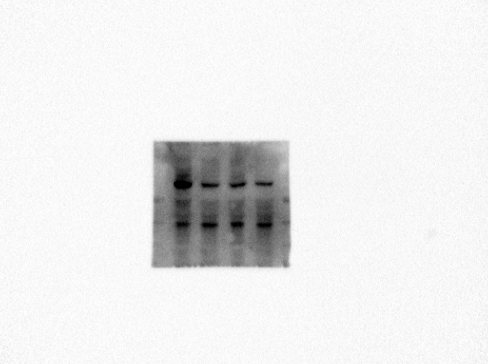

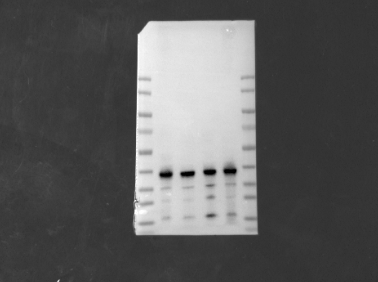


Figure 6G


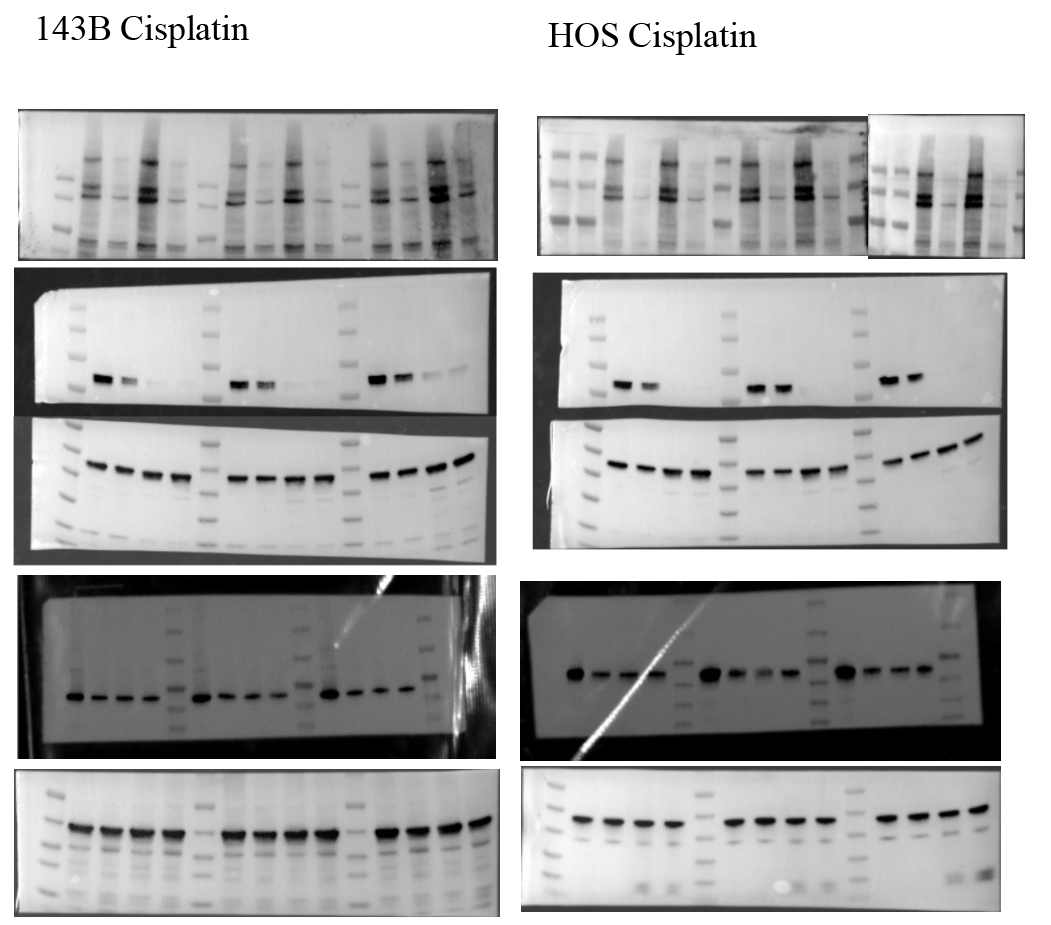


**Figure 7H**

143B


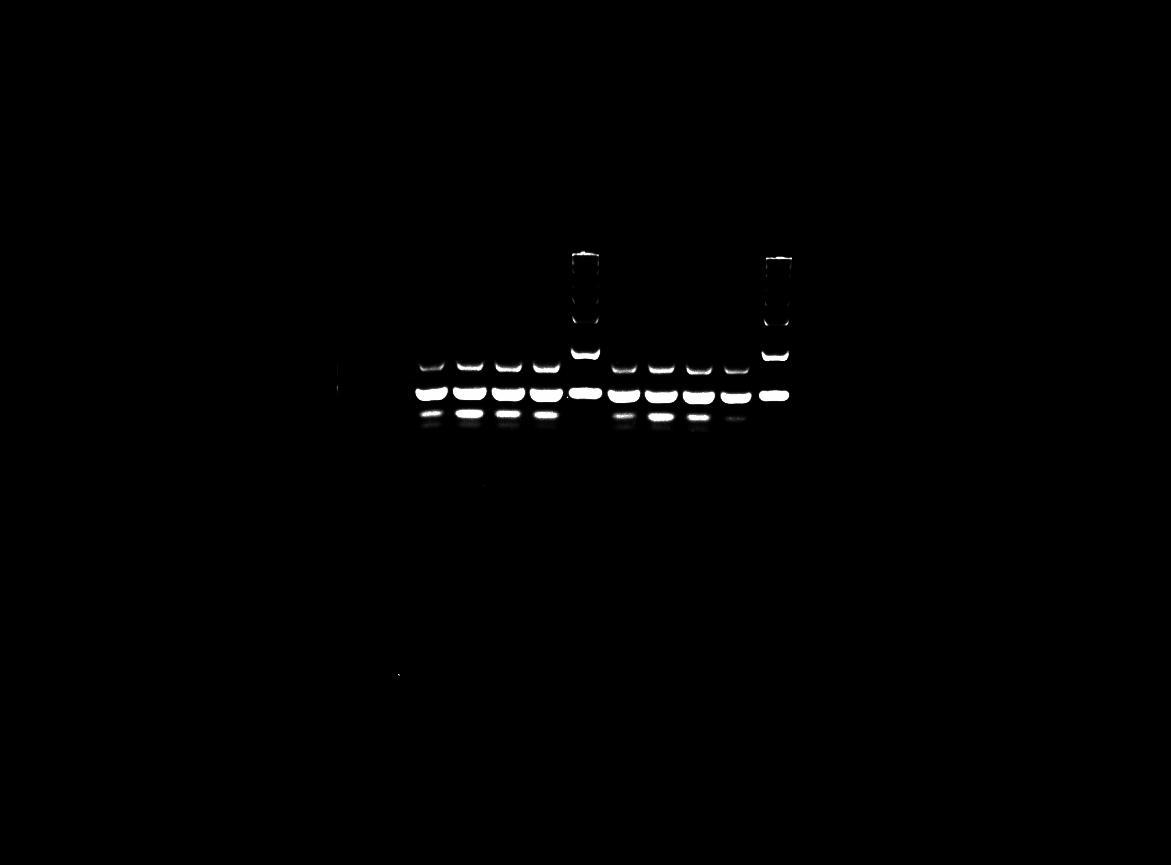


HOS


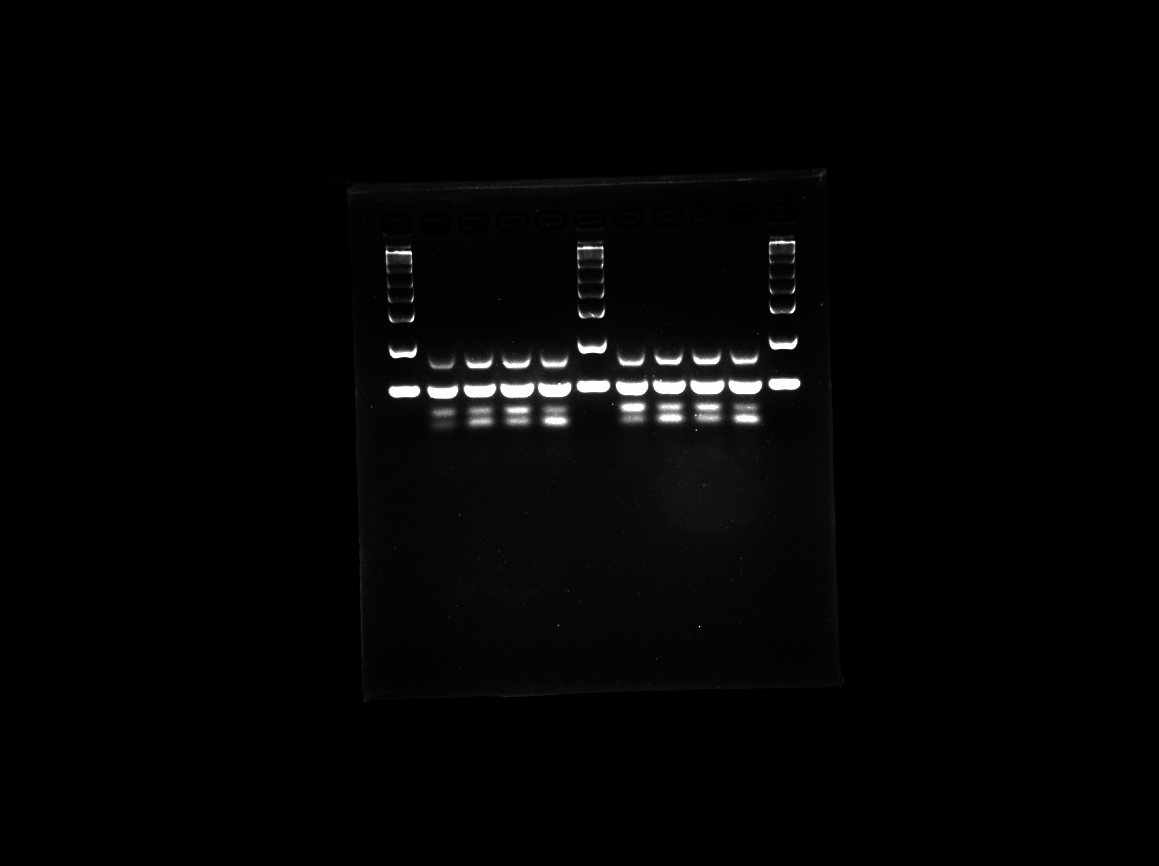


Figure 7I

143B


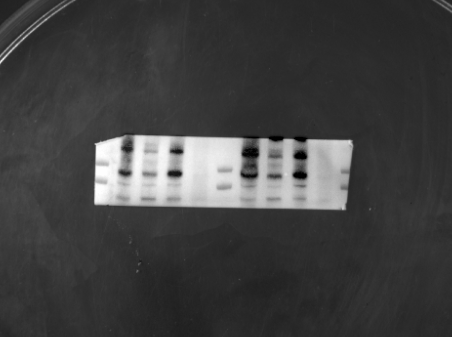

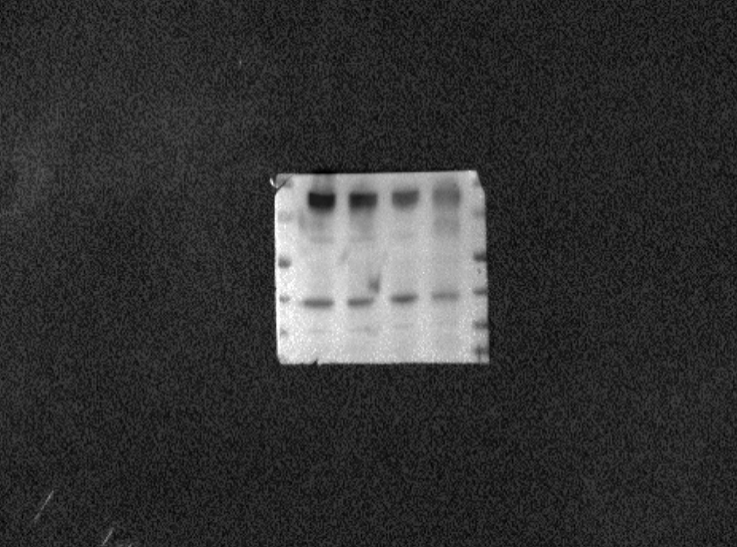

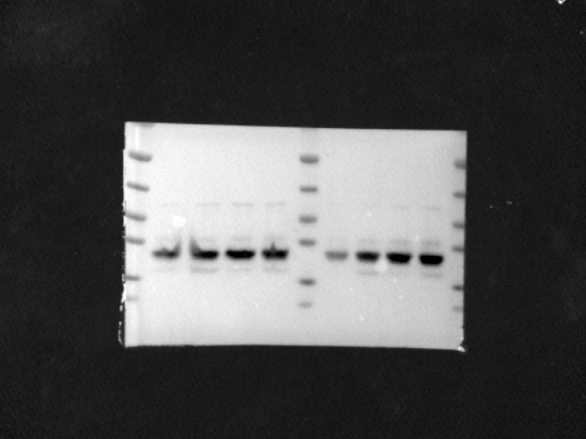

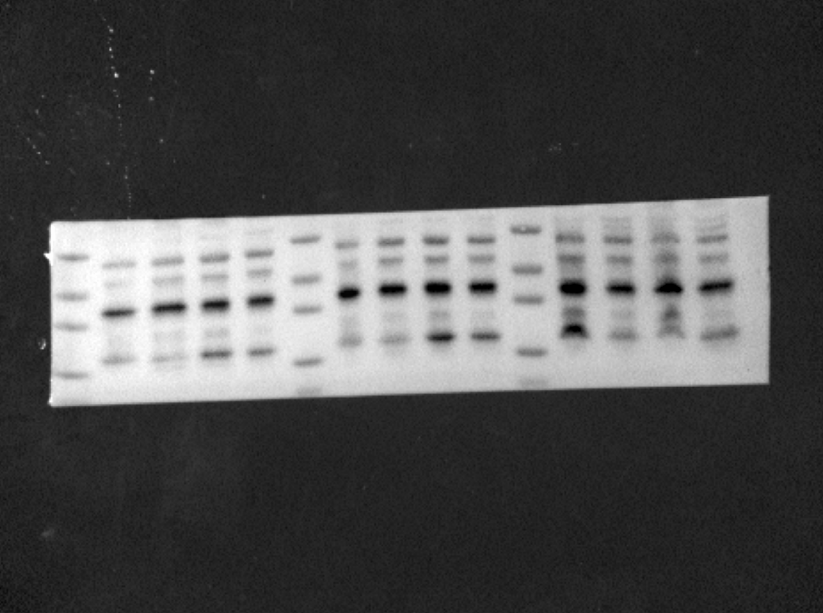

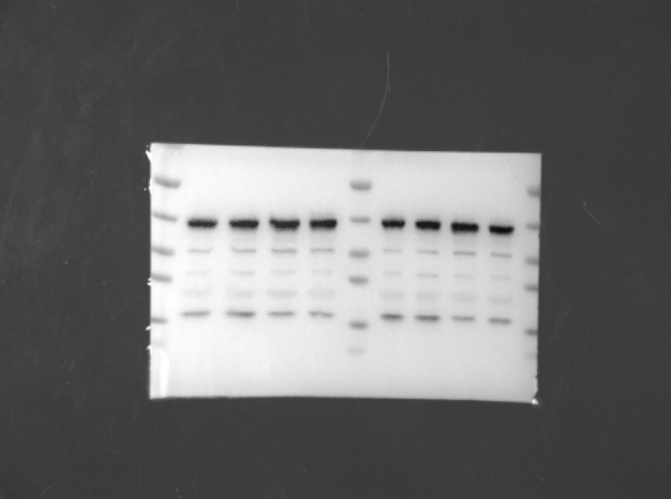


HOS


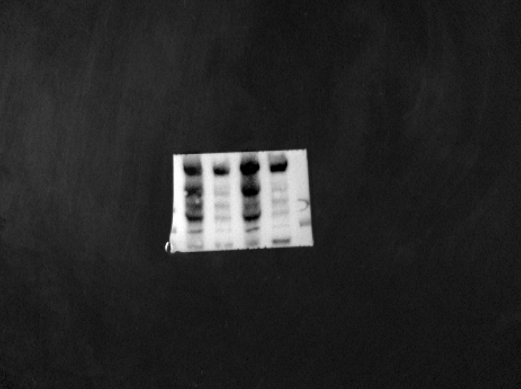

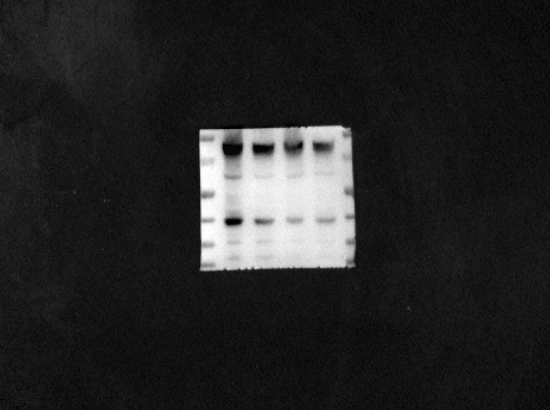

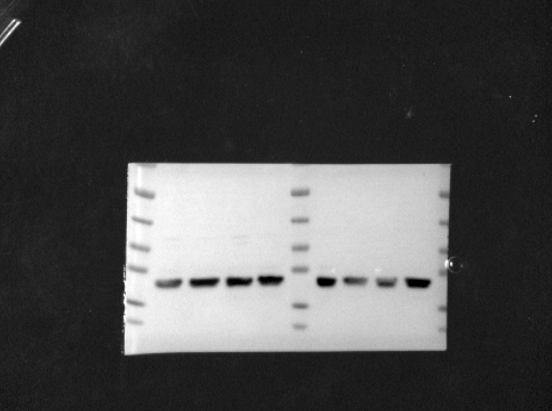

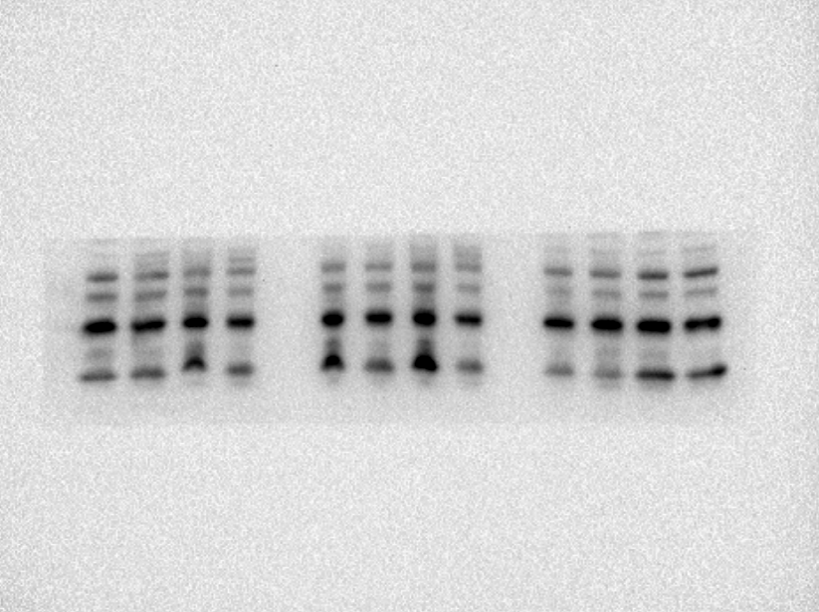

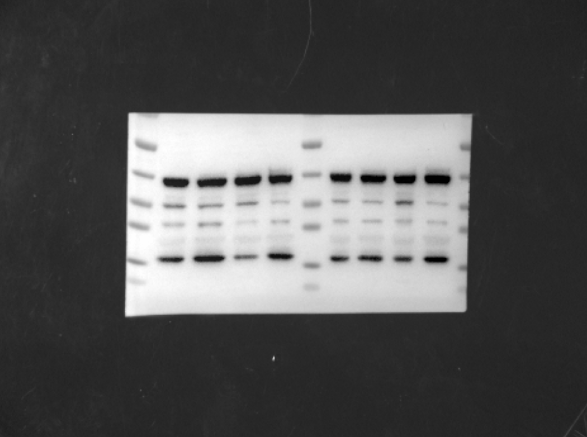

Supplement: Supplementary file 1 — Supporting Information [file ADVS-12-2500632-s005.docx]
